# Recombinant expression and subcellular targeting of the particulate methane monooxygenase (pMMO) protein components in plants

**DOI:** 10.1038/s41598-023-42224-9

**Published:** 2023-09-15

**Authors:** Tatiana Spatola Rossi, A. Frances Tolmie, Tim Nichol, Charlotte Pain, Patrick Harrison, Thomas J. Smith, Mark Fricker, Verena Kriechbaumer

**Affiliations:** 1 Endomembrane Structure and Function Research Group, Department of Biological and Medical Sciences, Oxford Brookes University, Oxford, OX3 0BP UK; 2 Molecular Microbiology Research Group, Biomolecular Sciences Research Centre, Sheffield Hallam University, Sheffield, S1 1WB UK; 3 Department of Biological and Marine Sciences, University of Hull, Hull, HU6 7RX UK; 4 Department of Biology, University of Oxford, Oxford, OX1 3RB UK; 5 Centre for Bioimaging, Oxford Brookes University, Oxford, UK

**Keywords:** Plant biotechnology, Plant cell biology

## Abstract

Methane is a potent greenhouse gas, which has contributed to approximately a fifth of global warming since pre-industrial times. The agricultural sector produces significant methane emissions, especially from livestock, waste management and rice cultivation. Rice fields alone generate around 9% of total anthropogenic emissions. Methane is produced in waterlogged paddy fields by methanogenic archaea, and transported to the atmosphere through the aerenchyma tissue of rice plants. Thus, bioengineering rice with catalysts to detoxify methane *en route* could contribute to an efficient emission mitigation strategy. Particulate methane monooxygenase (pMMO) is the predominant methane catalyst found in nature, and is an enzyme complex expressed by methanotrophic bacteria. Recombinant expression of pMMO has been challenging, potentially due to its membrane localization, multimeric structure, and polycistronic operon. Here we show the first steps towards the engineering of plants for methane detoxification with the three pMMO subunits expressed in the model systems tobacco and Arabidopsis. Membrane topology and protein–protein interactions were consistent with correct folding and assembly of the pMMO subunits on the plant ER. Moreover, a synthetic self-cleaving polypeptide resulted in simultaneous expression of all three subunits, although low expression levels precluded more detailed structural investigation. The work presents plant cells as a novel heterologous system for pMMO allowing for protein expression and modification.

## Introduction

Methane (CH_4_) is a potent greenhouse gas, and the second largest contributor to global warming after CO_2_. Globally, over 60% of methane emissions come from human activities, including industrial gas and petroleum systems, agricultural livestock, rice agriculture, and landfills^1,2^. Of those, rice agriculture contributes to around 9% of anthropogenic methane emissions^3^. In paddy fields, methane is produced in the soil due to the anoxic environment created by flooding, which favours the growth of methanogenic archaea, and the presence of organic matter derived from root exudates^4^. The rice plants themselves play a major role in funnelling emissions from the soil to the atmosphere as the gas is absorbed through the roots and transported upwards through porous aerenchyma tissue, leading to high internal methane levels^5^. For example, the concentration of methane in the medullar cavity of field grown rice was found to be 2900 times that of ambient air^6^. Hence, bioengineering strategies to engineer plant systems for methane detoxification are of great interest. This can include rice for paddy field emissions but also plants in general to populate landfill sites and wetlands.

### Nature’s predominant methane catalyst: pMMO

In nature, methanotrophic bacteria comprise the only terrestrial sink of methane gas. They inhabit the oxic layer of methane-rich soils such as landfills and paddy fields and use methane as their sole source of carbon and energy^7^. Methanotrophic bacteria break down methane due to the presence of methane monooxygenase enzymes (MMOs), specifically particulate methane monooxygenase (pMMO) and soluble methane monooxygenase (sMMO), which catalyse the reaction from methane to methanol^8–10^:

$${\text{CH}}_{{4}} + {\text{O}}_{{2}} + {\text{2e}}^{ - } + {\text{2H}}^{ + } \to {\text{CH}}_{{3}} {\text{OH}} + {\text{H}}_{{2}} {\text{O}}.$$

sMMO is a cytoplasmic multi-subunit enzyme complex and is the least abundant MMO, expressed only in some species and in the presence of low copper-to-biomass ratios^11^. Due to its cytoplasmic nature, it is relatively easier to manipulate than pMMO and therefore has been characterised in more detail. However, recombinant expression of active sMMO in a non-methanotroph has not been achieved to date^10^. Previously five of the sMMO proteins were expressed in Arabidopsis plants, with the hypothesis that expression of sMMO in plants grown to a large biomass (i.e. all transgenic maize in the US) could produce significant detoxification of atmospheric methane^12^. However, methane oxidation activity of sMMO-expressing Arabidopsis plants was not detected.

Recent mathematical modelling for pMMO suggests that expression of this enzyme complex in rice plants could effectively reduce methane emissions from the crop^13^. pMMO is the predominant MMO found in nature, present in almost all species of methanotrophic bacteria. pMMO is a copper-dependent integral membrane protein complex composed of three different subunits (PmoC, PmoA and PmoB) which are encoded in an operon^11^. Crystallographic data shows that the subunits assemble to form a heterotrimer of approximately 100 kDa which is repeated three times to form a nonamer^14,15^.

### Engineering and expression of pMMO in plant cells

Expression of pMMO in heterologous systems has been challenging in the past, most likely due to the complex membrane structure and localization of the enzyme, its copper requirement and (in cells other than bacteria) the fact that the genes encoding it form a polycistronic operon. In addition, the mechanisms of copper placement into the pMMO complex are not known. Recombinant expression of the full pMMO operon has so far only been achieved in the bacterium *Rhodococcus erythropolis,* albeit with levels of enzymatic activity 330 times lower than the methanotroph control^16^. Expression of the full-length PmoB subunit alone and its truncated variants in copper tolerant *E. coli* (K12 TB1) showed no methane oxidation activity for any PmoB variants^17^.

Plant cells could constitute a feasible vehicle to express pMMO due to the presence of complex subcellular membrane compartments that allow recombinant protein expression. In particular, the endoplasmic reticulum (ER) is the largest membrane compartment in the cell, and plays a major role in membrane protein synthesis and folding^18^. Moreover, the ER structure is highly dynamic and shows considerable plasticity in response to environmental changes and to varying protein production levels, contributing to the organelle’s capacity to harbour large amounts of foreign proteins^19,20^.

It is also reasonable that the copper requirement for pMMO can be met in plant systems. In plants, copper is an essential micronutrient, as it is present as a cofactor in enzymes required for photosynthesis, respiration, ethylene sensing, and oxidative stress response, amongst others^21^. The average composition of copper in leaves is between 5 and 20 mg g^-1^ dry weight^22^, with concentrations varying among plant species and varieties. Copper is required in multiple locations within the plant cell such as the cytosol, the mitochondrial inner membrane, the chloroplast stroma, the thylakoid lumen, the apoplast and the ER^23^. Copper transporters are present in the plasma membrane to uptake the metal, and in other organelles such as the ER. In particular, the ER contains the RAN1 copper transporter^21,24^, which supplies copper to the membrane-bound ethylene receptor^21^. Thus, it is feasible that sufficient copper ions would be available in the ER membrane for pMMO, depending on expression levels. In the case of requiring higher copper levels to support high pMMO expression, the use of copper tolerant rice strains could be considered^25^.

In terms of the downstream processing of methanol following methane oxidation, plants are reported to be resilient to certain levels of methanol^26^, and naturally produced methanol exits plant leaves through stomata where it is partially consumed by methanol-utilizing bacteria on the leaf surface^27,28^. In addition, although plants do not possess known enzymes to catalyse methanol oxidation, labelled methanol has been found to be partly converted to CO_2_ and organic molecules in various plant species^29,30^.

In this work, we take first steps towards creating such methane-detoxifying plants and show the possibilities of plants as a novel heterologous system for pMMO expression and protein engineering. The advantages of the ER membrane system for recombinant protein expression are utilised and pMMO expression is shown in the model plants *Nicotiana tabacum* (tobacco) and *Arabidopsis thaliana*. We found that all three subunits can be expressed and targeted to the ER in plant cells. Moreover, the subunits showed correct membrane topologies as well as protein–protein interactions. Applying a self-cleaving peptide system, all subunits were co-expressed without causing impact on ER structure and dynamics or plant health.

## Results

### Subcellular localization of recombinant pMMO proteins in tobacco

The native pMMO proteins PmoC, PmoA, and PmoB localize to the bacterial inner membrane in methanotrophic bacteria, with all three subunits featuring multiple transmembrane domains. For expression in plant cells, the likely subcellular localization of each subunit was initially predicted in silico using DeepLoc 1.0^31^. The prediction yielded a probability of almost 1 of all three proteins being folded as membrane proteins in Eukarya (Supplementary Figure S1A), and a high probability for localization of PmoC and PmoA to the ER, and for localization of PmoB to mitochondria or the ER (Supplementary Fig. S1B).

**Figure 1:**
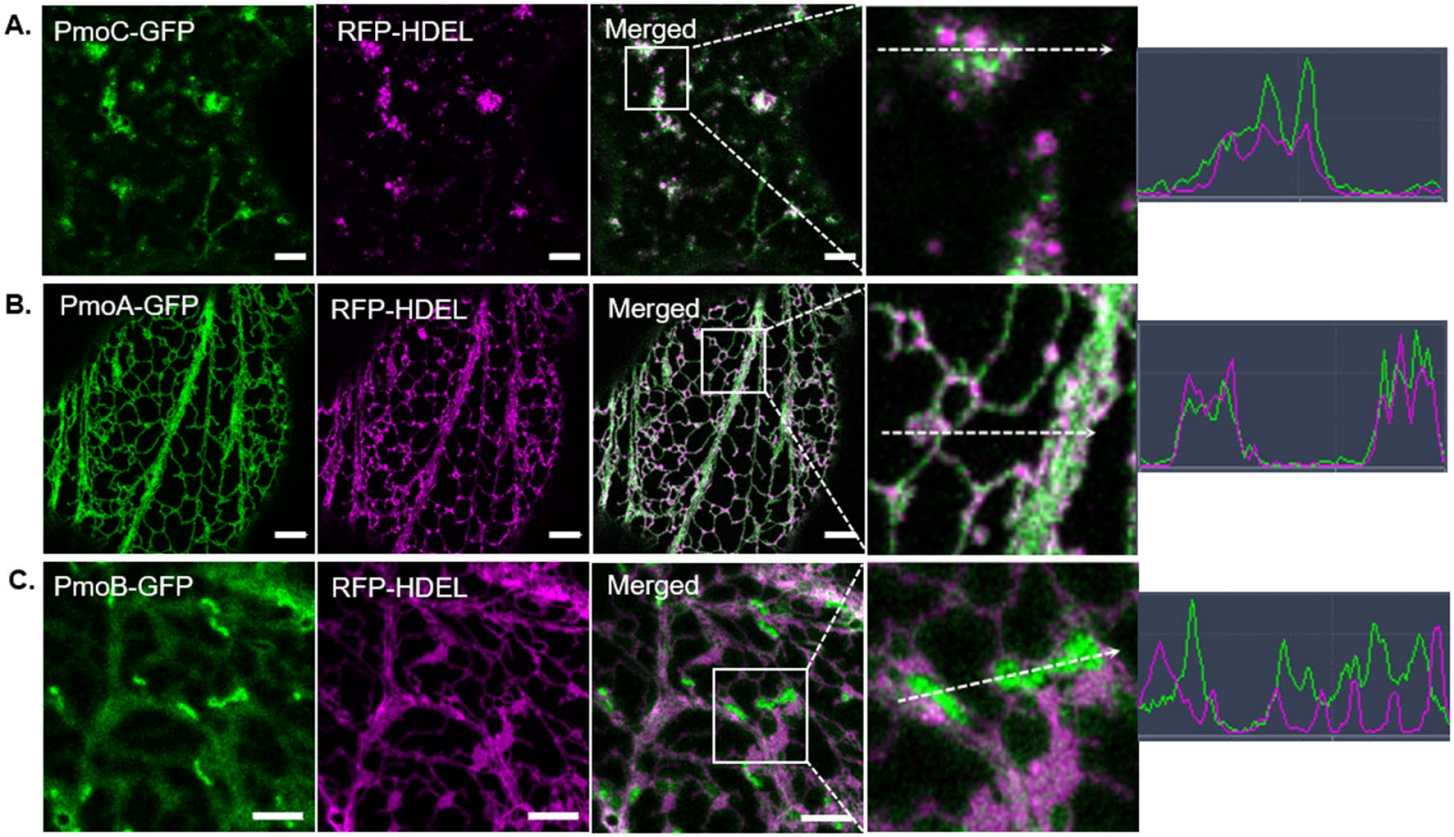
Subcellular localization of native pMMO proteins in transient expression in tobacco leaf epidermal cells. PmoC-GFP (**A**), PmoA-GFP (**B**) and PmoB-GFP (**C**) were expressed transiently in tobacco leaf epidermal cells together with the ER lumenal marker RFP-HDEL via Agrobacterium-mediated protein expression. Images were taken 2 days after infiltration (dai). pMMO subunits are shown in green, RFP-HDEL in magenta. White boxes in the merged channel indicate the enlarged area used for line profile co-localization analysis. White dotted arrows show the line along which a line profile was drawn plotting the fluorescence intensity of the green and red channels. Scale bars = 5 μm.

To analyse subcellular localization of the pMMO subunits in plant cells, the individual pMMO subunits were cloned into binary plant expression vectors under a strong constitutive promoter (35S), fused to a GFP tag, and transiently co-expressed in tobacco via agroinfiltration. Cells were imaged using confocal microscopy with an Airyscan detector for enhanced resolution. As predicted in silico, PmoC and PmoA fused with GFP localized to the ER and showed co-localization with the ER marker RFP-HDEL^32^ (Fig. 1A,B). PmoA did not cause any visible impact on the ER structure. However, PmoC disrupted the ER network structure.

Expression of PmoB-GFP revealed a combination of cytosolic signal and discrete brighter punctate objects (Fig. 1C). A line profile encompassing some puncta shows a negative correlation between the puncta (green) and the ER signal (magenta) (Fig. 1C), suggesting that these puncta do not reside in the ER. As PmoB was predicted to localize to mitochondria or ER, PmoB-GFP was co-expressed with the mitochondrial marker RFP-ATPase and showed colocalisation with the mitochondrial marker (Supplementary Fig. S2).

### Targeting PmoB to the ER

To assemble the pMMO enzyme complex, all subunits needed to be localized to the same membrane compartment. The PmoB subunit contains an N-terminal signal peptide which directs the protein to the periplasm in bacteria^33^, and could potentially be causing the observed mitochondrial localization in plants. Therefore, PmoB was targeted to the ER by replacing its bacterial signal peptide with a plant signal peptide from pumpkin (Cucurbita sp) 2S albumin, yielding csPmoB_40–431_. This signal peptide is generally cleaved from the proteins it targets, which in principle would preserve the native N-terminus of PmoB that is a ligand of one of the copper centres in the homologous pMMO from *Mc. capsulatus* Bath^34^. Predictions using SignalP6.0 showed a likelihood of 0.9998 of the recombinant protein containing a signal peptide in Eukarya, and a probability of 0.976649 of the protein containing a cleavage site. It is therefore highly likely that cs acts as a signal peptide in plant cells when fused to PmoB and becomes efficiently cleaved, as is the case when targeting native plant proteins^35^. Indeed, csPmoB_40–431_-GFP was successfully targeted to the ER (Supplementary Fig. S3A), however, the expression levels in the ER were very low. This could be due to the protein escaping the ER and being trafficked to downstream compartments in the secretory pathway. Specifically, transport of csPmoB_40–431_-GFP to the apoplast or vacuole would result in no visible fluorescence of the GFP fluorophore due to the acidic pH and proteolysis^36–38^. To test this, a fusion protein was created with an RFP moiety (csPmoB_40–431_-RFP), which can be detected in apoplast and vacuole. csPmoB_40–431_-RFP localized both to the vacuole and to the ER at 2 days after infiltration, and entirely to the vacuole at 3 days after infiltration (Supplementary Fig. S3B).

To avoid protein escaping to the vacuole, the addition of an ER retention tag was investigated. The HDEL retention tag is present in soluble proteins which are retained in the ER lumen, whereas the dilysine motif (KKRY) is present in the cytosolic tail of some type I membrane proteins^39,40^. It was expected that the csPmoB_40–431_ termini would be located in the ER lumen; therefore, the HDEL tag would produce retention whereas the dilysine motif would not. Indeed, csPmoB_40–431_-RFP-HDEL localized entirely to the ER at both 2 and 3 days after infiltration (Supplementary Fig. S3C). The dilysine motif did not induce retention, showing vacuolar localization at 2 and 3 days (Supplementary Fig. S3D) similarly to the construct without a retention tag. This showed that adding the HDEL tag permitted RFP-tagged and ER-localized PmoB with enhanced expression levels. In addition, it confirmed that the C-terminus of csPmoB_40–431_-RFP is localized in the ER lumen.

### Membrane topology of the pMMO subunits in plant cells

**Figure 2:**
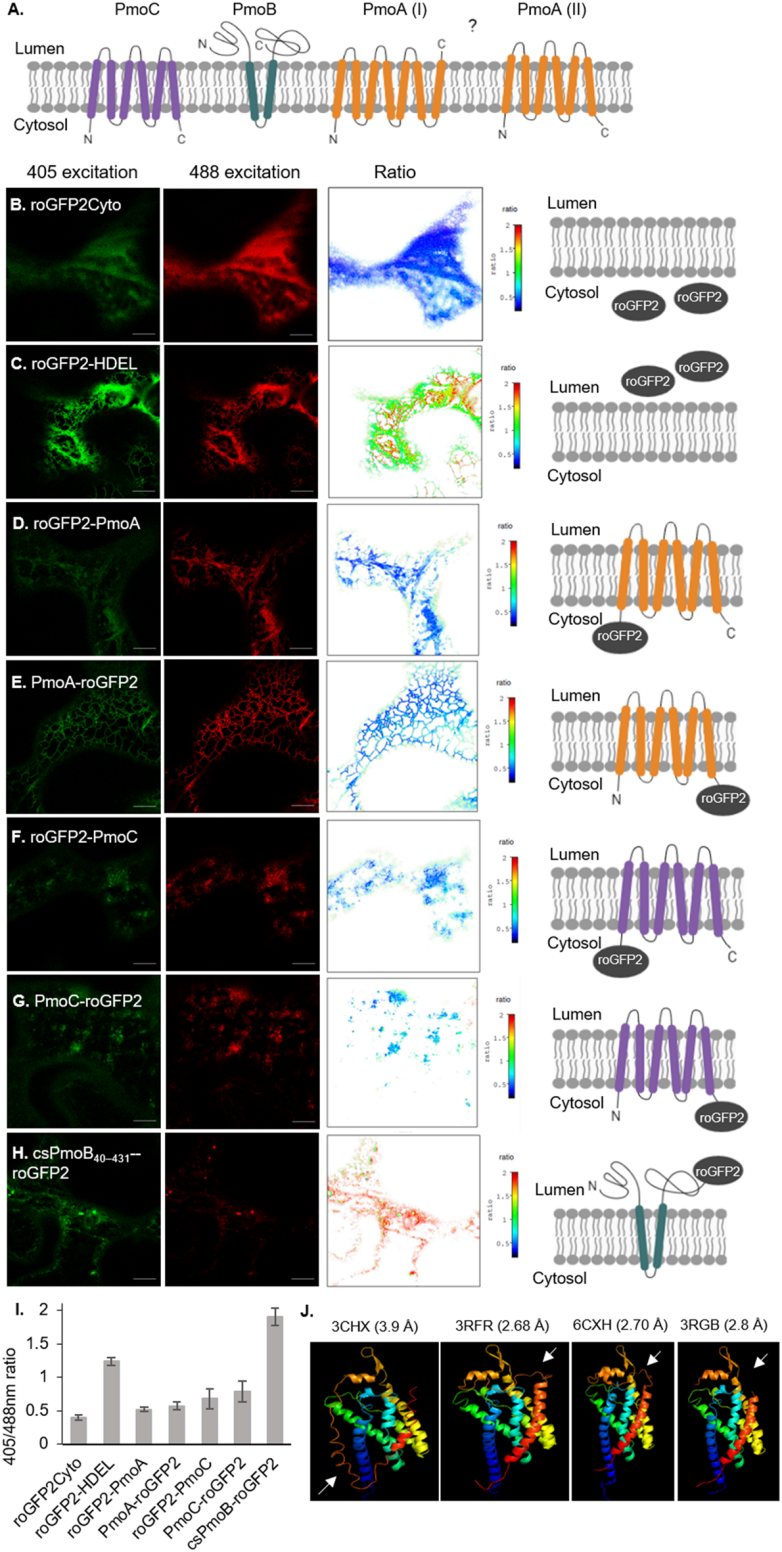
Membrane topology of pMMO proteins on the ER. (**A**) Membrane topology of PmoC and PmoB on the ER according to information from the crystal structure^15^ and computational predictions^41^. Two membrane topologies of PmoA are possible: PmoA (I) is supported by the crystal structure (3CHX), PmoA (II) is supported by computational predictions. Confocal images and pixel by pixel ratio of (**B**) roGFP2Cyto, (**C**) roGFP2-HDEL, (**D**) roGFP2-PmoA, (**E**) PmoA-roGFP2, (**F**) roGFP2-PmoC, (**G**) PmoC-roGFP2 and (**H**) csPmoB_40–431_-roGFP2. Scale bar = 10 μm. (**I**) Bar chart showing the mean 405/488 nm ratio and standard deviation for each construct, data taken from n = 3 with cells ≥ 8. (**J**) Crystal structure of PmoA from *Methylosinus trichosporium* OB3b (PDB ID: 3CHX) and from other methanotroph species (*Methylocystis sp.* strain M (PDB ID: 3RFR), *Methylomicrobium alcaliphilum* 20Z (PDB ID: 6CXH), and *Methylococcus capsulatus* (Bath) (PDB ID: 3RGB). The loop that traverses the membrane in 3CHX but not in the other structures is indicated with a white arrow.

The membrane topology of the pMMO proteins on the ER was investigated to assess whether the subunits are correctly oriented with respect to each other. Crystallography data from *Methylosinus trichosporium* OB3b (PDB ID 3CHX^15^) shows that the PmoC subunit contains at least four transmembrane domains, although the 80 C-terminal amino acids are unmodelled^15^. However, bioinformatics tools (CCTOP^41^) consistently predicted two additional transmembrane domains in the unmodelled region of PmoC, yielding a total of six transmembrane domains, with both termini facing the cytosol (Supplementary Fig. S4; Fig. 2A). Both the crystal structure and computational predictions show a topology of two transmembrane domains for PmoB with both termini facing the lumen (Fig. 2A). For the PmoA subunit, the crystal structure (3CHX) shows six transmembrane domains and an additional loop region that traverses the membrane, yielding a topology where the N-terminus of PmoA is located in the cytosol and the C-terminus is located in the lumen^15^. Computational predictions of PmoA were inconsistent in the number of transmembrane domains assigned (varying between five and seven transmembrane domains) with the CCTOP consensus showing six transmembrane domains and both termini facing the cytosol (Fig. 2A; Supplementary Fig. S4).

In order to test the PmoA topology in plant cells, a redox-based method was used based on fusion with the redox-sensitive GFP protein (roGFP2) on either protein terminus^42,43^. The constructs roGFP2-PmoA and PmoA-roGFP2 were cloned and transiently expressed in tobacco leaf epidermal cells. The controls roGFP2Cyto and roGFP2-HDEL were used for measurements of the cytosol and ER lumen, respectively. Cells were imaged by confocal microscopy with excitation both at 405 and 488 nm. The ratio of roGFP2 excitation at 405–488 nm gives information regarding the redox state of the protein environment: a low 405/488 ratio indicates a reducing environment such as the cytosol, whereas a higher 405/488 ratio indicates an oxidative environment such as the ER lumen^44^. Images were processed using redox ratio analysis software which performs background subtraction and smoothing, and calculates a pseudo color coded pixel by pixel ratio^45^. As expected, roGFP2Cyto was excited with a higher efficiency at 488 nm (405/488 ratio = 0.41 ± 0.04; Fig. 2B,I), whereas roGFP2-HDEL was excited with a relative higher efficiency at 405 nm (405/488 ratio = 1.24 ± 0.06; Fig. 2C,I). roGFP2-PmoA (405/488 ratio = 0.52 ± 0.03; Fig. 2D,I) and PmoA-roGFP2 (405/488 ratio = 0.57 ± 0.06; Fig. 2E,I) both had a higher excitation at 488 nm, similar to roGFP2Cyto, indicating that both PmoA termini are facing the cytosol. This PmoA topology aligns with crystal structures of PmoA from other genera of methanotrophs, which have both termini facing the cytosol, as a result of a loop being modelled differently to the 3CHX structure and not traversing the membrane (Fig. 2J).

roGFP2 fusions to PmoC yielded a disrupted ER network, however, the images and ratio values reflected the predicted topology with both termini facing the cytosol (405/488 ratio = 0.68 ± 0.15 for roGFP2-PmoC and 0.79 ± 0.15 for PmoC-roGFP2; Fig. 2F,G,I). A roGFP2 fusion to the C-terminus of csPmoB_40–431_ showed an ER lumenal localization (405/488 ratio = 1.9 ± 0.13; Fig. 2H,I).

### Protein–protein interactions of pMMO subunits on the ER membrane

**Figure 3:**
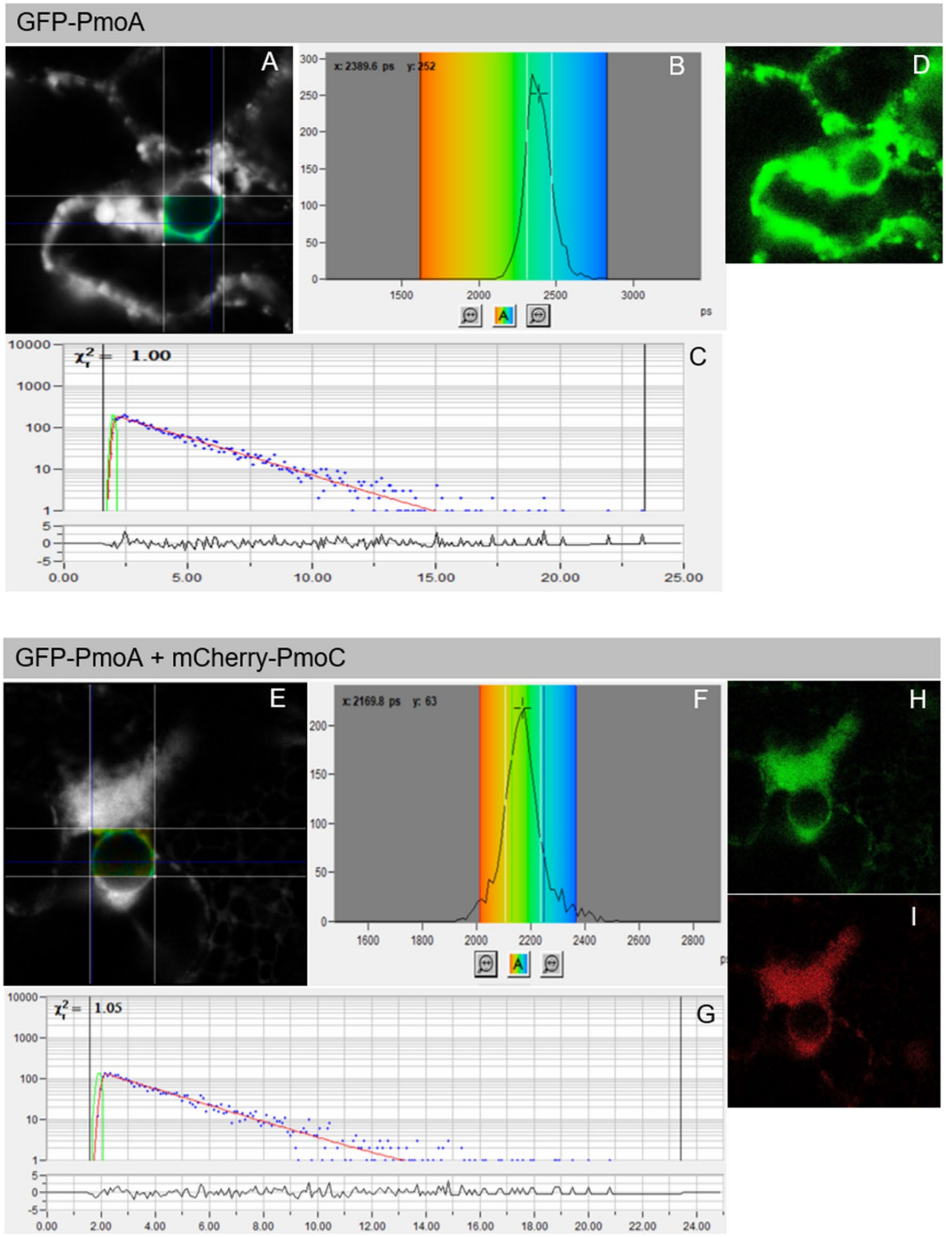
FRET-FLIM analysis of GFP-PmoA without an interaction partner (**A**–**D**) or with mCherry-PmoC (**E**–**I**). (**A** and **E**) display the raw FRET-FLIM data and pseudocolored lifetime maps showing the lifetime values for each point within the region of interest. (**B** and **F**) show the distribution of lifetimes across the image, with blue shades representing longer GFP fluorescence lifetimes than green ones. (**C** and **G**) display representative decay curves of a single point with an optimal single exponential fit, where x^2^ values from 0.9 to 1.2 were considered an excellent fit to the data points (binning factor of 2). The inset in (**D**), (**H**) and (**I**) are the respective confocal images for the analysis, showing the GFP construct in green and the mCherry construct in red. This example of FRET-FLIM analysis shows that GFP-PmoA interacts with mCherry-PmoC, because the lifetime values for the GFP/mCherry fusion pair (H; 2.2 ns) are significantly lower than those for the GFP fusion alone (**D**; 2.4 ns).

The native pMMO complex is a nonamer with three repetitions of the PmoC, PmoA and PmoB trimer^14^. Successful reconstitution of the complex requires *in planta* interactions mainly between PmoC and PmoA, and between PmoA and PmoB, which are tightly associated in the crystal structure^14^. Förster resonance energy transfer by fluorescence lifetime imaging (FRET-FLIM) was used to assess the presence of pairwise protein–protein interactions on the ER membrane. FRET-FLIM is based on measuring a reduction in the fluorescence lifetime of the donor molecule (GFP) in the presence of an acceptor (RFP). In the case of an interaction, the donor and acceptor fluorophores are required to be at a close enough distance (< 10 nm) for energy to be transferred from the donor to the acceptor molecule. This transfer of energy results in a decrease in the fluorescent lifetime of the donor. Due to the ER being a highly dynamic organelle, FRET-FLIM was carried out in the nuclear envelope, which is contiguous with the ER membrane but has a relatively low mobility, to give more reliable measurements^32,46^ (Fig. 3).

**Figure 4:**
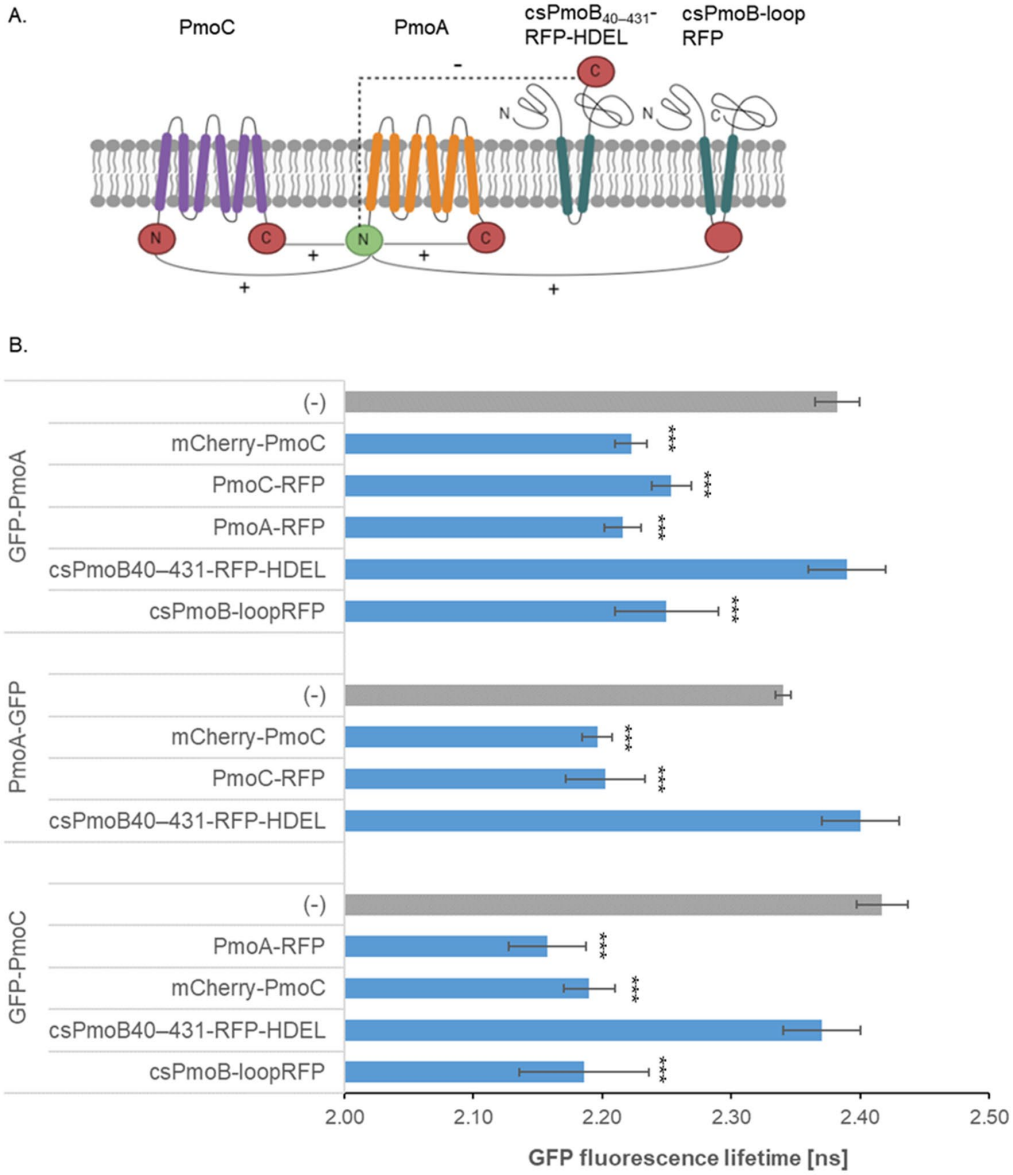
Fluorescence lifetimes in FRET-FLIM interactions. (**A**) Schematic diagram of the interactions of GFP-PmoA (donor molecule, green), with the different acceptors (red), interactions ( +) and no interactions (-) found via FRET-FLIM are shown. (**B**) The bar graphs represent average fluorescence lifetimes [ns] and the corresponding SD values for the GFP donors GFP-PmoA, PmoA-GFP and GFP-PmoC. The data show the donor lifetimes without interaction partners (dark grey bars) compared to the lifetimes co-expressed with pMMO subunits (blue bars). Significance was analysed by Kruskal–Wallis (***p < 0.001). Lifetimes significantly lower than those of GFP-PmoA, PmoA-GFP and GFP-PmoC alone indicate protein–protein interactions. n = 4 with at least 6 cells per combination.

A significant reduction in the lifetime of GFP-PmoA was found in the presence of mCherry-PmoC and PmoC-RFP (reductions of 0.16 ns and 0.13 ns, respectively, Figs. 3, 4; Supplementary Table 1), indicating an interaction between the N-terminus of PmoA and both termini of PmoC. An example image of GFP-PmoA without interaction partner as negative control and with the interaction partner mCherry-PmoC is shown in Fig. 3, representative images and raw data from the remaining combinations are shown in Supplementary Fig. S5. Protein–protein interactions were also found between PmoA-GFP and PmoC tagged on either terminus further confirming the topology for both proteins.

PmoC and PmoA did not interact with csPmoB_40–431_-RFP-HDEL (Fig. 4B) which is consistent with the opposite facing topology of these proteins that brings donor and acceptor fluorophores on opposite sides of the membrane (Fig. 4A). To analyse protein–protein interactions between PmoB and the other subunits, csPmoB_40–431_-RFP-HDEL was modified to now feature the fluorophore in the PmoB loop region between the two TMDs (between residues 233 and 234). In the construct csPmoB_-_loopRFP the fluorophore is now facing the cytosol. csPmoB_-_loopRFP showed protein interactions with PmoA and PmoC (Fig. 4; Supplementary Table 1). Homotypic interactions were also tested and showed all subunits capable of interacting with themselves (Fig. 4; Supplementary Table 1).

### Gene stacking using 2A self-cleaving peptides

The pMMO proteins are encoded in an operon in bacteria^11^. Transient expression of the three subunits from individual plasmids in tobacco leaf cells resulted in less than 1 in 1000 cells expressing all three proteins. In order to employ fewer number of co-transformations, a method based on binary plant “2in1” vectors was used which contain two cloning cassettes within the same T-DNA region^47^. However, dual expression from the vectors containing pairs of pMMO genes was not observed. Instead, only one cassette was seen to express from each construct or, in some cases, neither was expressed (Supplementary Fig. S6).

**Figure 5:**
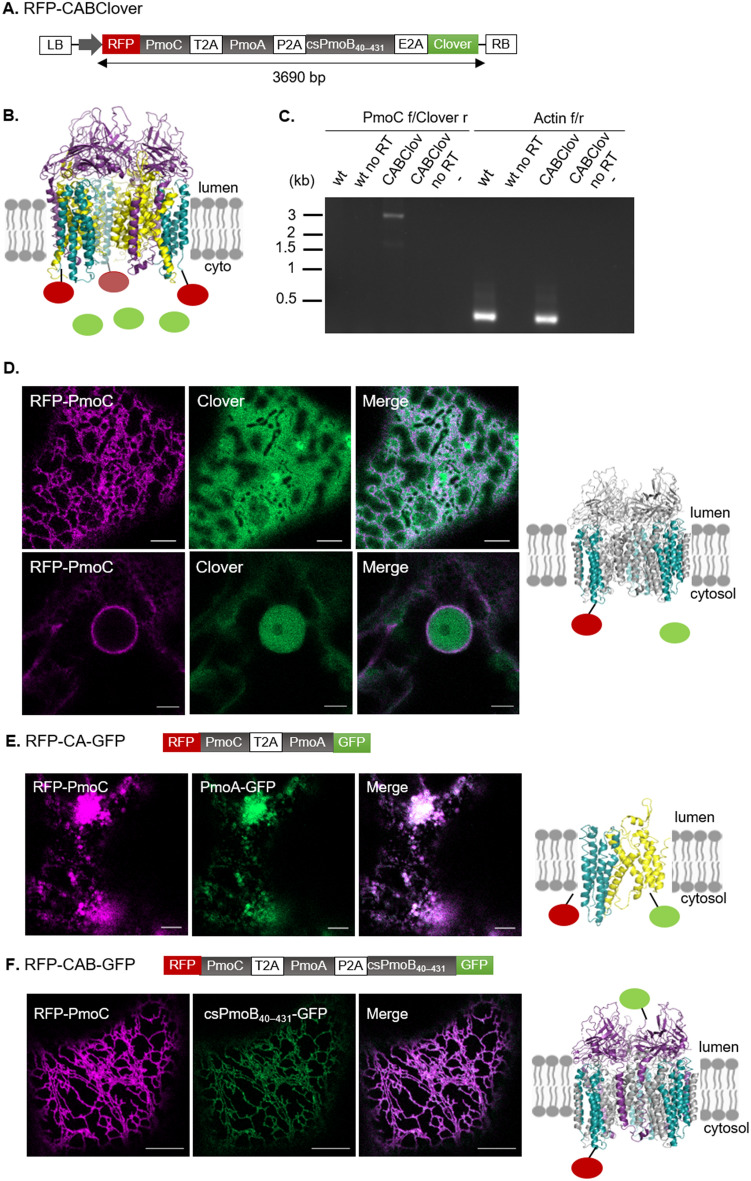
Co-expression of pMMO proteins using 2A self-cleaving peptides. (**A**) Schematic diagram of RFP-CABClover containing the three subunits and Clover separated by three self-cleaving peptides (T2A, P2A and E2A). LB and RB = left and right border of T-DNA region. Grey arrow = promoter. (**B**) Diagram showing the expected assembly of pMMO on the ER membrane and localization of fluorophores following translation of the polypeptide. (**C**) RT-PCR after transient transformation of tobacco (2 days after infiltration) with RFP-CABClover. RNA was extracted from a leaf piece in the infiltrated area (RFP-CABClover) or from a non-infiltrated leaf piece (wt). No RT controls for cDNA reactions without reverse transcriptase are shown. PCR was carried out for the polypeptide transcript (expected size 3251 bp) and with actin primers (expected size 435 bp). (**D**) Transient expression of RFP-CABClover showing localization of RFP-PmoC in the ER and of Clover in the cytosol, with highlight of the differential localization of RFP-PmoC in the nuclear envelope and of Clover in the nucleoplasm. (**E**) Transient expression of RFP-CA-GFP showing localization of both RFP-PmoC and PmoA-GFP in the ER, with perturbation to ER morphology. (**F**) Transient expression of RFP-CAB-GFP showing localization of both RFP-PmoC and csPmoB_40–431_-GFP in the ER. Scale bar = 5 μm.

Subsequently, an alternative gene stacking method based on “2A” self-cleaving peptides was applied. Self-cleaving peptides are short virus-derived sequences which produce cleavage of polyproteins by a ribosomal skipping mechanism during translation between the last and second to last residues in the sequence^48^. A quad-cistronic polypeptide was designed containing all pMMO proteins separated by different self-cleaving peptides followed by a terminal Clover fluorophore (Fig. 5A). The Clover fluorophore allows screening of cells for expression but was not fused to the protein structure as it is separated via a self-cleaving peptide. The choice and order of self-cleaving peptides was based on the reported highest efficiency for expression of quad-cistronic vectors using 2A peptides: in the order T2A, P2A, and E2A^49^. The gene order for pMMO was maintained as it is present in the bacterial operon (*pmoCAB*), as previous studies have found that the gene order within an operon tends to be optimized for complex assembly in multimeric protein complexes, with proteins adjacent to each other in an operon being more likely to interact^50^. The polypeptide was fused N-terminally to RFP yielding RFP-CABClover (Fig. 5A), to show translation at the N-terminus of the polypeptide and subcellular localization of the PmoC subunit. The N-terminus of PmoC in the crystal structure is in the cytosolic side and faces the outside of the structure; therefore, it is likely that the fluorophore addition would not greatly impact the enzyme structure or assembly (Fig. 5B).

RFP-CABClover transcription was assessed via RT-PCR. A band of the expected size (3,251 bp) was detected (Fig. 5C; Supplementary Fig. S7). Transient expression at 2 days after infiltration showed cells producing RFP-PmoC in the ER and Clover in the cytosol (Fig. 5D). This indicated that PmoC was correctly targeted to the ER and that the E2A peptide was cleaved yielding the cytosolic localization of the Clover fluorophore. The distinct subcellular localization of both proteins is highlighted by the labelling of the nuclear envelope by RFP-PmoC and of the nucleoplasm by Clover (Fig. 5D). Further constructs were created to determine the subcellular localization of PmoA and csPmoB_40–431_ within the polypeptide using double fluorophore fusions: RFP-CA-GFP and RFP-CAB-GFP. With RFP-CA-GFP, both RFP-PmoC and PmoA-GFP localised to the ER, with most cells showing disruption of the ER network (Fig. 5E). For RFP-CAB-GFP, both RFP-PmoC and csPmoB_40–431_-GFP labelled the ER, and expression of all three subunits appears not to perturb the ER network (Fig. 5F). Taken together, this showed that all three subunits can be co-expressed and sequentially targeted to the ER in tobacco using self-cleaving peptides.

Expression levels of the polypeptide following transient transformation of tobacco were low. Therefore, RFP-CABClover was co-expressed with P19, a suppressor of post transcriptional gene silencing (PTGS)^51^ in *Nicotiana benthamiana.* Expression with P19 resulted in a 3.1-fold increase of RFP-PmoC and a 2.3-fold increase of Clover (Supplementary Fig. S8), indicating that PTGS is fully or partly responsible for the polypeptide’s low expression in plants.

### Impact of the pMMO polypeptide on ER structure and dynamics

Maintaining the cell’s natural ER architecture is critical for a bioengineering strategy where the pMMO enzyme needs to function alongside normal cell physiology. Large natural cell-to-cell variation exists in ER structure, i.e. in the proportion of the two main structural features: tubules and cisternae (Supplementary Fig. S9A–C); hence the AnalyzER software^52^ was used to quantitatively detect changes in ER structure and dynamics of cells expressing the pMMO polypeptide (RFP-CAB) or the individual pMMO subunits.

Tobacco cells expressing the lumenal marker GFP-HDEL were used as a measure of wild-type ER, (Supplementary Fig. S9D). The PmoC subunit was not included in this analysis as cells, which express just PmoC, present a disrupted ER network (Fig. 1A). Fluorescence intensity levels were quantified to ensure that cells with similar expression levels between constructs were used (Supplementary Fig. S9E).

Expression of PmoA showed a constriction phenotype with the lumenal marker GFP-HDEL in thinner tubules interspaced with bright bulges (Supplementary Fig. S10A–E), resembling the constriction phenotype induced by overexpression of some reticulon proteins^53,54^. A skeleton could not be extracted consistently from the GFP channel with co-expression of PmoA-RFP for software analysis and PmoA-RFP was not further analyzed.

**Figure 6:**
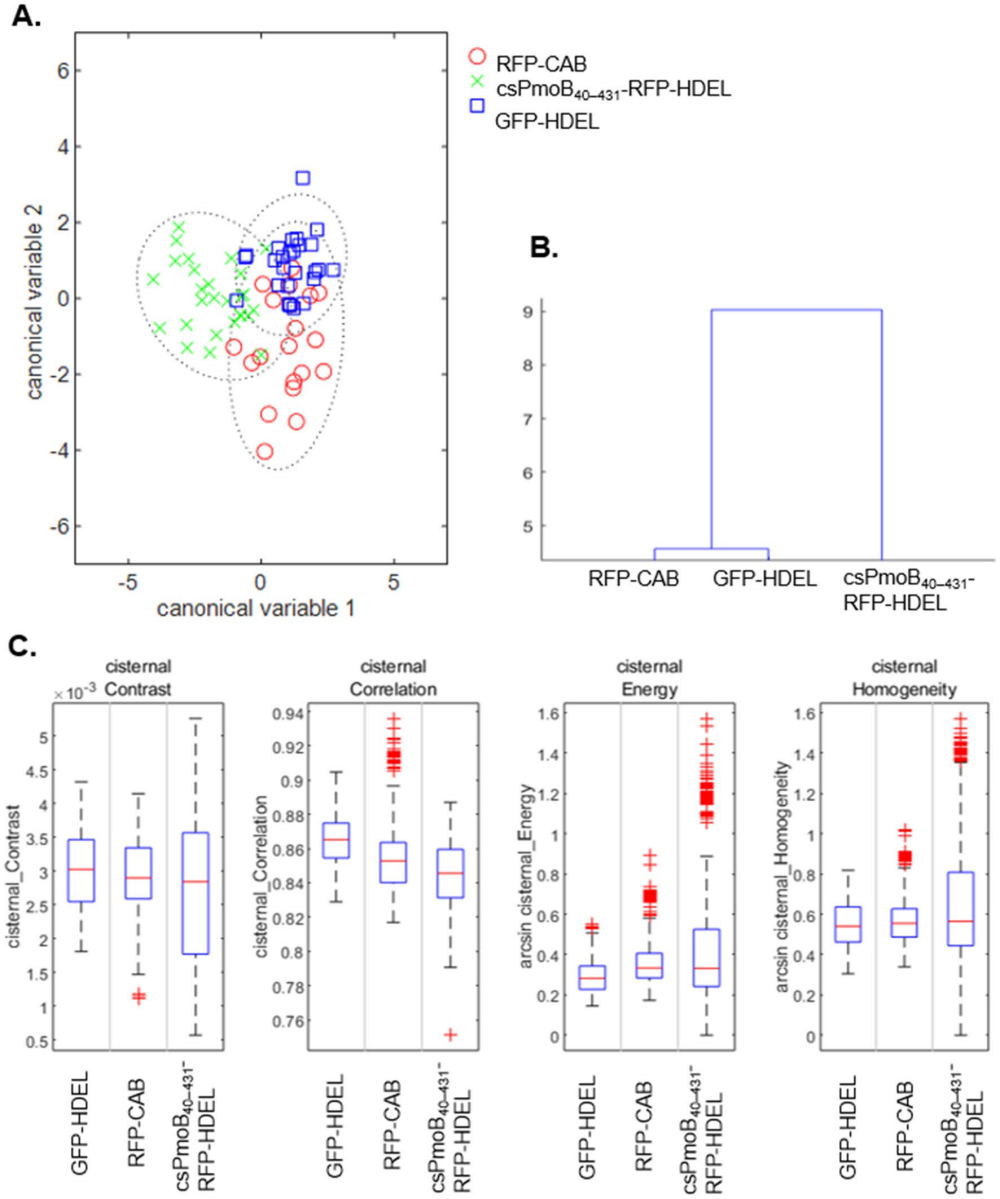
Multivariate analysis of ER structure and dynamics. Analysis conducted on AnalyzER with metrics extracted from 30-frame time series at 821 ms intervals, in cells expressing RFP-CAB + GFP-HDEL (n = 20), csPmoB_40–431_-RFP-HDEL + GFP-HDEL (n = 27), and GFP-HDEL alone (n = 26). (**A**) MANOVA using 19 metrics of ER structure and dynamics showing a significant difference between the groups (Pillai’s trace, F(3.227), p = 1.5 × 10^–6^; Roy’s largest root, F(5.7947), p = 2.39 × 10^–7^). Dotted lines represent the 95% confidence limit. (**B**) Dendrogram showing the clustering based on the groups’ multivariate means. (**C**) Metrics of cisternal texture show a wide variation of csPmoB_40–431_-RFP-HDEL for cisternal contrast, cisternal energy and cisternal homogeneity, and a significantly lower cisternal correlation of RFP-CAB and csPmoB_40–431_-RFP-HDEL with the GFP-HDEL control.

The impact of the expression of csPmoB_40–431_-RFP-HDEL and RFP-CAB upon ER architecture (Supplementary Fig. S10F-K) was compared to GFP-HDEL alone using a multivariate analysis of variance (MANOVA), combining 19 metrics of ER structure and dynamics^52^. The MANOVA showed significant differences in the ER architecture between the groups, with the three groups being separated with two canonical variables (Fig. 6A). A dendrogram plotting the groups’ multivariate means shows a very close similarity between the network of cells expressing RFP-CAB and the GFP-HDEL control, and a larger separation of csPmoB_40–431_-RFP-HDEL (Fig. 6B). Post-hoc comparisons showed a significant difference in cisternal correlation, a metric of cisternal texture, between RFP-CAB and GFP-HDEL, and between csPmoB_40–431_-RFP-HDEL and GFP-HDEL (Supplementary Table 2). This indicated that RFP-CAB and csPmoB_40–431_-RFP-HDEL had less homogeneous cisternae than the control (Fig. 6C). Significant differences were also detected between csPmoB_40–431_-RFP-HDEL and GFP-HDEL in cisternal maximum speed and between csPmoB_40–431_-RFP-HDEL and both other groups in cisternal mean persistency, with csPmoB_40–431_-RFP-HDEL presenting a slightly lower maximum speed and higher persistency of cisternae (Supplementary Table 2, Supplementary Fig. S11A). No significant differences were found between the various remaining metrics, such as those of cisternal or polygonal region morphology, or tubule length (Supplementary Table 2, Supplementary Fig. S11B and C). These results suggest that the expression of the RFP-CAB polypeptide does not induce major changes to the ER structure or dynamics. Moreover, it induces less ER perturbations than the single subunits expressed individually, with csPmoB_40–431_-RFP-HDEL showing the least impact, followed by PmoA, which causes a constriction phenotype, and then by PmoC, which induces severe ER disruption.

### Testing pMMO enzyme activity in plant extracts

Infiltrated leaves transiently expressing the pMMO polypeptide RFP-CABClover in tobacco were used to perform enzyme activity assays. Two additional constructs were created to test whether the addition of fluorophores or residues to the pMMO proteins would impede enzymatic activity: CABClover, which is lacking the N-terminal RFP fused to PmoC, and RFP-CABint featuring an intein sequence that removes self-cleaving peptide residues attached to the C-terminal of PmoA. *Nicotiana benthamiana* plants were infiltrated with either RFP-CABClover, CABClover, or RFP-CABint, each co-expressed with P19 to enhance expression levels. Infiltrated leaves were ground in non-denaturing extraction buffer and plant lysates were tested for activity by adapting the methane monooxygenase assay using the surrogate substrate propylene that is used to assay methanotroph cells^55^ with a 0.08 µg ml^-1^ detection limit of the GC system for the assay product. Given the low level of expression of the pMMO proteins observed compared with those in methanotroph cells (where they are among the most highly expressed proteins), it is likely that the amount of the enzyme in the plant cells was not sufficient to produce detectable enzyme product (Supplementary Fig. S12). pMMO enzyme activity in methanotrophic bacteria whole cells has been calculated as 1.3 µg mg^-1^ dry weight min^-1^^16^ with pMMO accounting for approximately 20% of the total protein content. pMMO in plants is expressed at 2000 times lower levels (approximately 0.01% of total protein content). This would only provide an activity of 0.65 ng mg^-1^ dry weight min^-1^ in plants, which is below the method detection levels. Additionally, the assembly into a functional nonomer complex could be impaired and lead to a lack of enzymatic activity. Hence, future studies will require biochemical analysis on complex size and composition but for this, a higher yield is required.

### Stable expression of the pMMO polypeptide in Arabidopsis plants

**Figure 7:**
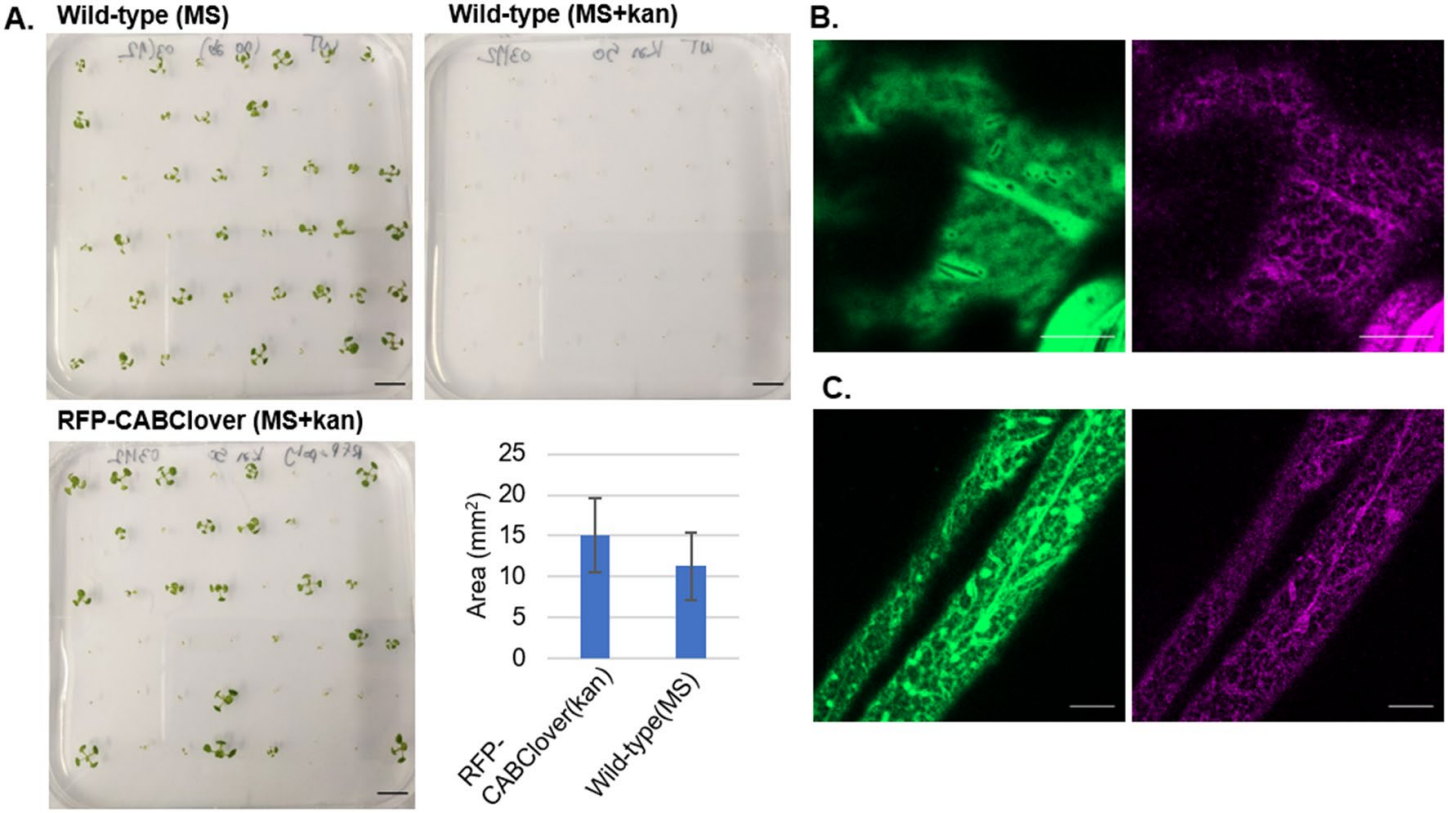
Stable Arabidopsis containing RFP-CABClover. (**A**) 10 day old Arabidopsis seedlings of T2 RFP-CABClover grown in selective media (MS + 50 mg L^-1^ kanamycin), and wild-type plants grown in non-selective media (MS) (scale bar = 10 mm), with quantification of seedling size showing the mean and standard deviation. Wild-type plants grown in selective media had 0% germination rate showing the efficacy of the antibiotic concentration used for selection. (**B**) 5 day old Arabidopsis T2 RFP-CABClover seedlings expressing Clover in the cytosol and trace levels of RFP-PmoC in the ER in cotyledons, and roots (**C**). Scale bars = 10 μm.

Arabidopsis plants expressing RFP-CABClover in a stable manner were generated in order to assess the viability of such plants. T2 seeds germinated in selective media and did not show apparent phenotypic defects (Fig. 7A). Quantification of the area of individual seedlings yielded an average area of 15 ± 4 mm^2^ for RFP-CABClover and 11 ± 4 mm^2^ for wild type seedlings. 5-day-old seedlings were imaged and showed expression of RFP and Clover in cotyledons (Fig. 7B) and root cells (Fig. 7C). In mature plants, only expression of Clover could be detected, suggesting a downregulation of the pMMO genes.

## Discussion

### Targeting of pMMO subunits to the plant ER

Despite the substantial biotechnological potential, recombinant expression of pMMO is largely unexplored due to the difficulties of heterologous expression^10,11,16,33^. In this work, the pMMO subunits were expressed in plant cells and fused to fluorescent proteins to assess subcellular localization. Transient expression in tobacco showed that PmoC and PmoA localized to the ER, without the addition of any targeting motifs. A major route for targeting proteins to the endoplasmic reticulum is the co-translational pathway, via the signal recognition particle (SRP)^56^. This transport process is highly conserved across the domains of life, with the ER membrane being analogous to the inner membrane in Gram-negative bacteria^56^. It is possible thus, that the PmoC and PmoA subunits are being targeted to the ER in plants through their first transmembrane domain in a similar way to their targeting to the bacterial inner membrane in methanotrophic bacteria. Interestingly, the PmoB subunit, which contains a bacterial signal peptide, localized to mitochondria in plant cells, indicating that the bacterial signal peptide is not recognized by the plant SRP. The majority of cells expressing PmoC showed disruption of the ER network, whereas cells were able to express PmoA and ER targeted PmoB (csPmoB_40–431_-RFP-HDEL) at high levels without disruption to the ER network. This indicated that tobacco cells are able to express all pMMO subunits individually, and that they can be targeted to the ER, but may cause disruption. In turn, the ER network architecture was not impaired considerably by the pMMO polypeptide RFP-CABClover. Moreover, it was shown that stable Arabidopsis plants expressing the polypeptide were able to germinate and grow without visible defects. The structure of the plant endoplasmic reticulum is naturally very plastic^57–59^ (and remains functional across a range of structures. Even somewhat extreme phenotypes, such as the pah-1 pah-2 mutant which exhibits vastly increased ER content per cell due to unrepressed phospholipid synthesis develop normally, if more slowly^60^. Therefore, perturbations of ER structure to the extent described on RFP-CAB over-expression are expected to be well tolerated by the plant. This is also supported by the development of a stable RFP-CABClover line.

### Topology and interactions of pMMO proteins on the ER membrane

To obtain functional pMMO, the native topology and interactions between the subunits must be reconstituted. The topology tested experimentally here using redox-sensitive GFP showed that both termini of PmoA localised to the cytosol *in planta*, differing from the 3CHX structure^15^, which showed the N-terminus facing the cytosol and the C-terminus facing the lumen, but in agreement instead with computational predictions and crystal structures from other methanotroph species^14,61,62^. CryoEM structures of the pMMO from *Methylococcus capsulatus* Bath reconstituted in lipid nanodiscs^34^ are also in agreement with the predictions of the topology of the *M. trichosporium* pMMO presented here in terms of the positions of the termini of PmoA relative to the membrane and the additional two transmembrane segments of PmoC. It is possible that errors in the modelling of a loop region (residues 196–220) in 3CHX led to a different topology.

Protein–protein interactions tested via FRET-FLIM also supported this topology, and showed pairwise interactions between all three subunits. This is consistent with complex assembly on the ER membrane but further work is required to determine whether the assembly is a functional nonomer with the native metal centres of pMMO.

### The use of 2A self-cleaving peptides for subunit co-expression

Gene stacking using multiple promoters can present problems such as gene silencing and varying stoichiometry^38,63,64^. Indeed, 2in1 vectors^47^ containing two cloning cassettes with tandem 35S promoters, resulted in no expression of one or both expression cassettes. Alternatively, 2A self-cleaving peptides have been used to co-express proteins in eukaryotic cells under a single promoter^65–67^. Here all pMMO proteins could be co-expressed via self-cleaving peptides under a single promoter, and be sequentially targeted to the ER.

### Steps towards a functional pMMO in plant cells for methane detoxification

This work showed that all three pMMO subunits can be expressed in the plant ER using self-cleaving peptides as a gene stacking method. pMMO subunits are capable of protein–protein interactions and with this complex formation, though it is unclear whether the complex has the structure and metal content of native pMMO. Plant cells as a novel recombinant expression system for pMMO allow for protein modifications and alternative targeting which can be of importance to investigate novel catalytic properties of this biotechnologically important enzyme complex.

Further work is required to explore the enzyme activity of the recombinant system. Currently protein extracts did not exhibit measurable activity with the commonly used sMMO/pMMO substrate propene^55,68,69^. This can be due to a number of factors including *in planta* complex formation, cofactors and reducing agents, assay detection limits or protein expression levels. In regards to cofactors, membrane preparations of methanotrophic bacteria exhibit NADH-driven pMMO activity, however, the reductase partner for pMMO in the native bacteria has not been conclusively defined, with synthetic quinols used as a reducing agent in vitro^55,68^. The plant ER features NADPH-dependent electron transport between cytochrome P450s and their reductases and oxidation substrates^70^, and the reduction of protein disulphides by ER oxidoreductases in a not fully characterised reduction pathway^71^. Therefore, the capacity of the ER environment to supply the electrons needed for pMMO turnover remains to be investigated. The plant protein extracts used for propene activity tests were therefore supplemented with duroquinol, which is known to be able to supply electrons to pMMO^72^. Since the aim of the work is to remove methane, assays with the less reactive substrate methane would also be needed.

Transient and stable expression of RFP-CABClover yielded less than 0.01% of total plant protein. pMMO enzyme activity from *M. trichosporium* has been quantified as 0.13 µg mg^-1^ protein min^-1^ in purified membrane fractions^15^ and 1.3 µg mg^-1^ dry weight min^-1^ in whole bacterial cells^16^. These activities are achieved with pMMO accounting for approximately 20% of the total bacterial protein and 80% of total protein in enriched membrane fractions^73^. Hence, the level of *in planta* pMMO production was identified as a major likely cause of any product from the recombinant enzyme failing to reach the 0.08 µg ml^-1^ detection limit of the GC. Increasing production of the pMMO subunits in the plant from the levels already obtained should therefore be a major focus of future work. In addition, cell lysis has been shown to drastically decrease the enzyme activity in methanotrophic bacteria; hence it will be of interest to assay pMMO activity *in planta*.

Taken together we show the suitability of plant cells as heterologous expression system for bacterial enzyme complexes such as pMMO with the plant ER as a membrane platform for these complexes.

Expression of the three pMMO subunits in the model systems tobacco and Arabidopsis using self-cleaving peptide technology highlights the first steps towards the engineering of plants for methane detoxification.

## Materials and methods

### Cloning of expression plasmids

*Methylosinus trichosporium* OB3b was grown and total DNA was prepared as described previously^74^. pMMO genes (accession number: U31650.2) were amplified by PCR using Q5 high-fidelity DNA polymerase (New England Biolabs). Primers were obtained from Eurofins Genomics. Genes of interest were cloned into the modified binary vectors pB7WGF2, pB7FWG2, and pB7RWG2, containing an N-terminal green fluorescent protein (GFP), a C-terminal GFP, and a C-terminal red fluorescent protein (RFP), respectively^75^, via Gateway technology (Invitrogen). Genes were cloned into psso1 and pcmo1 destination vectors for topology analysis^42^. The polypeptide sequence (CABClover) was codon optimized for tobacco and synthesized by Twist Bioscience. Expression plasmids were used to transform competent *Agrobacterium tumefaciens* cells (strain GV3101) via heat shock.

### Plant material and transient transformation of tobacco

For Agrobacterium-mediated transient transformation, 5 week old *Nicotiana tabacum* SR1 cv Petit Havana (here referred to as tobacco) grown in greenhouse conditions were used. Transformation was carried out following the protocol in^76^. In brief, 2 mL of overnight Agrobacterium liquid cultures were spun down at 2200* g* for 5 min and resuspended in 1 ml of infiltration buffer (5 mg mL^−1^ glucose, 50 mM MES, 2 mM Na_3_PO_4_.12H_2_O and 0.1 mM acetosyringone). A second wash step was performed by repeating the centrifugation and resuspension in 1 mL of fresh infiltration buffer. Cultures were diluted to OD_600_ 0.1 with infiltration buffer. A hypodermic needle was used to make a small puncture on the abaxial surface of the leaves and the cultures were infiltrated into the leaves by slowly discharging the suspension on the puncture whilst holding the upper side of the leaf. Plants were then watered and placed in tobacco growth chamber (23/20 °C, 12 h light/dark) until imaged by confocal microscopy (generally 48 h after infiltration). For experiments with the P19 construct, *Nicotiana benthamiana* was used instead of tobacco. Collection of plant material, complies with relevant institutional, national, and international guidelines and legislation.

### Subcellular localization of the pMMO subunits

A small infiltrated leaf piece (approximately 5 mm^2^) was cut with a scalpel and placed on a glass slide with the abaxial side facing up. Water was added and slides were imaged using a Zeiss LSM880 confocal microscope with an Airyscan detector using a PlanApo 63x/1.46 NA oil immersion objective. Images were taken in a dual track mode with line switching, with GFP excitation at 488 nm and detection at 495–550 nm, and RFP excitation at 561 nm and detection with a long pass filter at 570 nm. Line profiles were plotted using the ZEN blue software.

### Redox-based membrane topology analysis

roGFP2-PmoA and PmoA-roGFP2 were cloned and infiltrated into *Nicotiana benthamiana* with P19, and compared with roGFP2Cyto and roGFP2-HDEL controls^42,43^ which were infiltrated without P19, to obtain similar fluorescence intensity levels. Images were acquired using a Zeiss LSM880 with a PlanApo 63x/1.46 NA oil immersion objective, using multitrack mode with line switching. Excitation was produced at 405 nm and 488 nm with emission at 500–555 nm, and excitation at 405 nm with emission at 410–470 nm for autofluorescence. Images were processed using a redox ratio analysis software^45^.

### Subunit interactions using FRET-FLIM

Images were acquired as described in^77^, using a modified Nikon EC2 confocal scanning microscope which permitted two-photon fluorescence lifetime imaging^78^. Excitation was produced with a 920 nm laser light generated by a titanium sapphire laser, emitting 200 fs pulses at 76 MHz, and focused with a water immersion Nikon VC/1.2 NA objective. The emitted light was collected without descanning, and passed through a BG390 (Comar) filter before reaching the detector. The detector was linked to a time-correlated single-photon counting PC module (SPC830, Becker and Hickl) to generate the raw FRET-FLIM data. The data was analysed using SPCImage 5.1 Software (Becker and Hickl), where lifetime exponential decay curves were traced for every pixel in the region of interest, in this case the nuclear envelope. The average excited-state lifetime values were obtained for each nuclear envelope.

### RT-PCR

RNA was extracted from sections of infiltrated tobacco leaves at 2 days after infiltration (between 100 and 200 mg approximately), using the NEB Total RNA Miniprep Kit, following the manufacturer’s instructions, with additional gDNA removal performed by in tube digestion with DNase I. cDNA was synthesized using ProtoScript® II First Strand cDNA Synthesis Kit (New England Biolabs), according to the instructions in the kit, using 1 μl of oligo(dT) primer and 1 μl of random primers. The PCR was carried out using One*Taq®* DNA Polymerase (New England Biolabs) and bands were identified by gel electrophoresis (1% agarose gel) with 2 μl/100 ml SYBR Safe DNA Gel Stain (Invitrogen). Primers polyCshortF (ATTCGTTCGCCCCTGAGTTC) and polyCloverShortR (ATTATACTCCAGCTTGTGGCCCA) were used for amplification of polypeptide cDNA, and primers Actinfor (TGGAACTGGAATGGTTAAGGCTGG) and Actinrev (TCTCCAGAGTCGAGCACAATACCG) were used for actin.

### ER structure analysis (AnalyzER software)

Image acquisition was carried out as described in^52^ using the Zeiss 880 PlanApo 100x/1.46 NA oil immersion objective, 2-line averaging, and Airyscan processing (0.09 μm/pixel and 0.82 ms frame time). 30-frame time series were acquired with dual track excitation at 488 nm (GFP) and 561 nm (RFP), with the GFP channel being imported into the AnalyzER software. Images were processed following the guidelines in the manual^52^, and using a min A cut-off value for the classification of cisternae of 1 μm^2^, and a lag value for persistency of 7 (equivalent to 5.7 s). The resulting data was processed using the AnalyzER statistics package based on MATLAB (R2020b). For the tubule trace analysis higher magnification images were used (0.02 μm/pixel), and both channels (GFP and RFP) were imported into AnalyzER.

### Production of stable Arabidopsis plants

*Arabidopsis thaliana* (Columbia-0) (here referred to as Arabidopsis) was transformed via the floral dipping method^79^. Briefly, Agrobacterium cultures were grown in LB containing antibiotics at 28 °C and 180 rpm overnight. The cultures were centrifuged at 2200* g* for 10 min. The pellets were resuspended in a 5% sucrose, 500 µl L^−1^ Silwet 77 solution. Arabidopsis plants at flowering stage were immersed in the solution for 30 s with agitation and placed in a tray covered with cling film to maintain humidity for 24 h. The plants were then watered and kept in greenhouse conditions and the floral dip was repeated a week later to increase the transformation efficiency. After a week of watering, the plants were bagged and left to dry for seed production. Selections were carried out on agar plates with 50 mg/L kanamycin. Collection of plant material, complies with relevant institutional, national, and international guidelines and legislation.

### pMMO enzyme activity assays

2 g of infiltrated *Nicotiana benthamiana* leaves per construct were ground to a fine powder in liquid nitrogen and lysed with non-denaturing extraction buffer using a mortar and pestle (10 mM Tris–HCl pH 7.5, 150 mM NaCl, 0.5% v/v Nonidet, and 1 mM serine protease inhibitor (PMSF, BioChemica), protease inhibitor cocktail (Sigma-Aldrich)). CuSO_4_ was added to the extraction buffer as copper addition is known to enhance enzymatic activity of pMMO in assays with methanotroph cells^80^. In methanotrophs, varying amounts of Cu^2+^ are added to the culture medium to allow pMMO expression and loading of the copper sites, such as 10–20 μM^81^, 40 μM^55^, 50 μM^15^. Additional copper is also often supplemented to assays. 40 μM CuSO_4_ was added to the lysis buffer following the conditions in^55^ where pMMO was purified in its active form. Lysates were placed on ice for 15 min and spun down at 2000 rpm for 2 min. 0.5 ml of dense supernatant was placed in a gas tight 1 ml vial (Sigma-Aldrich) containing duroquinol as a synthetic reducing agent for pMMO^68^. Duroquinol was made as in^72^. Duroquinol was added in excess as a solid at the bottom of the vial before adding the plant lysate. 0.7 ml of air was removed using a syringe and 1 ml of propene gas was added. Samples were incubated in a water bath at 30 °C for either 10 or 30 min. A negative control without propene was included for each sample. Vials were cooled on ice and extracted with 200 μl of toluene (Fisher Chemical).

### Detection by GC–MS

The activity of pMMO was tested by assaying the conversion of propene to propylene oxide^55^ using gas chromatography-mass spectrometry (GC–MS),comprising of a 7890A GC system coupled to a 5975C VL MS detector (Agilent Technologies) and fitted with a Poraplot Q column (25 m × 0.25 mm, Agilent Technologies). The Injection temperature and MS temperature were 250 °C. The column temperature was held at 180 °C for 8 min, then ramped up to 250 °C at 50 °C min^−1^ and held for 5 min. The flow rate of carrier gas (nitrogen) was 1 ml min^−1^, and the split ratio was 50:1. Detection was carried out using selected ion monitoring at 58.1 m/z corresponding to propylene oxide.

### Supplementary Information

Supplementary Information.

## Data Availability

Data is included as supporting information. Raw data is available from the corresponding author upon reasonable request. U31650.2 (*Methylosinus trichosporium* particulate methane monooxygenase operon).
