# Supplementary material for: Recombinant expression and subcellular targeting of the particulate methane monooxygenase (pMMO) protein components in plants
Source: Sci Rep. 2023 Sep 15;13:15337. doi: 10.1038/s41598-023-42224-9 (PMC10504283; doi:10.1038/s41598-023-42224-9)
Supplement: Supplementary file 1 — Supplementary Information. [file 41598_2023_42224_MOESM1_ESM.docx]

**Supplementary Data**


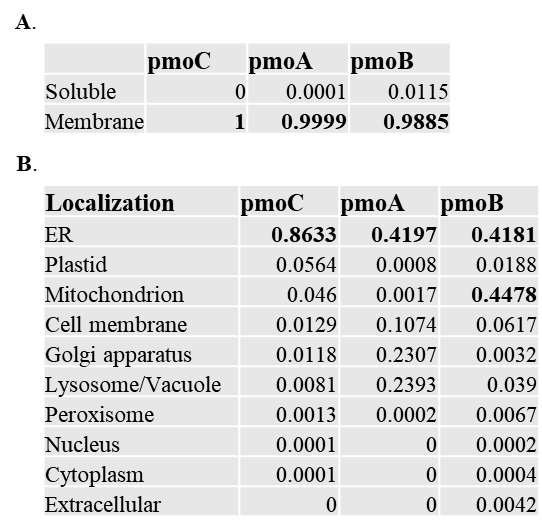


**Supplementary Figure S1. Subcellular localization predictions of pMMO proteins in Eukarya.**

(A) Probability of folding as a membrane protein in Eukarya using DeepLoc 1.0, (B) Subcellular localization prediction of pMMO proteins in Eukarya using DeepLoc 1.0 showing the probability calculated for each of 10 different cellular compartments.


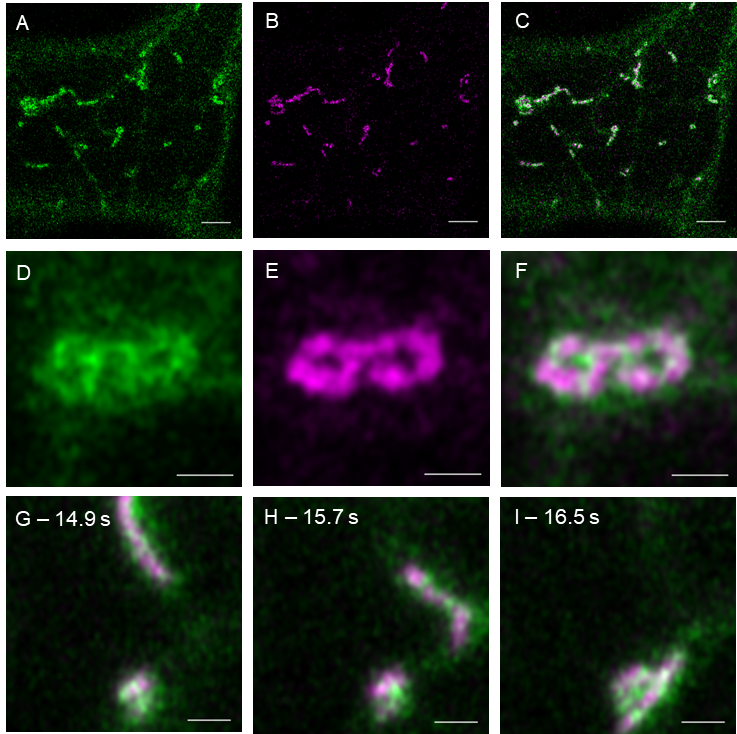


**Supplementary Figure S2. Co-localization of PmoB-GFP with mitochondria marker.**

PmoB-GFP (green) co-expressed with RFP-ATPase (magenta) appear to co-localize at low magnification (A-C). Scale bar = 5 μm. Co-localization was confirmed using higher magnification imaging (D-F). The analysis of a time series also supports the co-localization of PmoB-GFP with RFP-ATPase as both proteins move together (G-I). Scale bar = 1 μm.


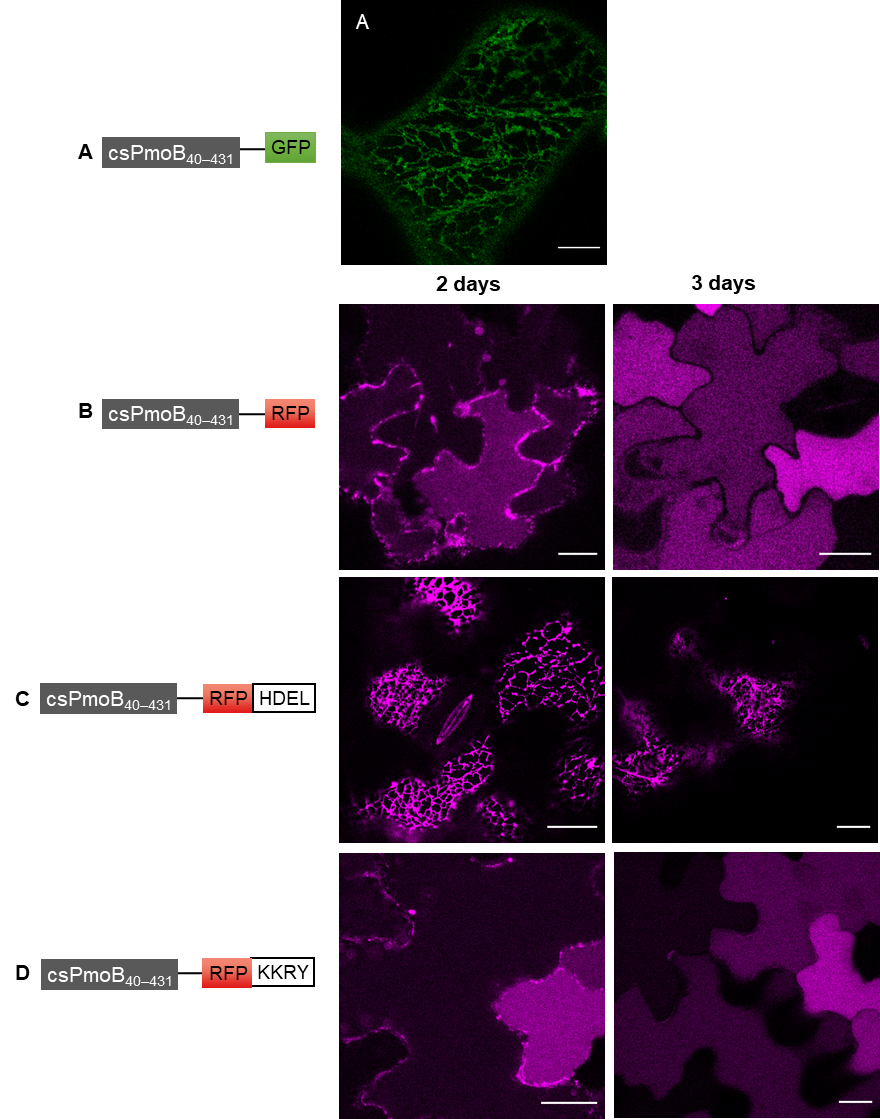


**Supplementary Figure S3. Targeting PmoB to the ER.**

(A) csPmoB_40–431_-GFP, in which the bacterial signal peptide of PmoB is replaced by a plant signal peptide, is successfully targeted to the ER. Scale bar = 10 μm (B) csPmoB_40–431_-RFP shows dual localization in the ER and vacuole at 2 days after infiltration (2 dai) and entirely vacuolar localization at 3 dai. (C) csPmoB_40–431_-RFP-HDEL retains the protein in the ER at both 2 and 3 days. (D) csPmoB_40–431_-RFP-KKRY shows similar localization pattern than the construct without any retention tags. Scale bars = 20 μm.


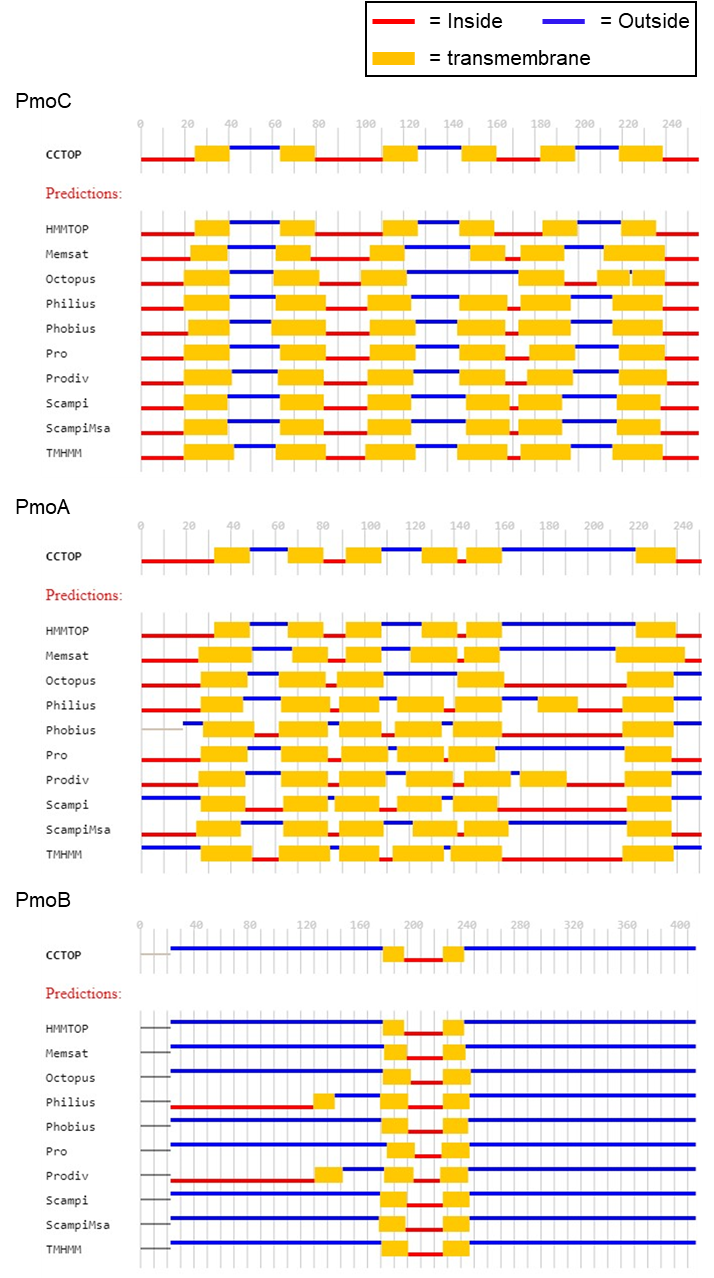


**Supplementary Figure S4. Prediction of transmembrane domains of the pMMO proteins from *Methylosinus trichosporium* OB3b.**

Computational prediction of transmembrane domains (yellow rectangles) using the CCTOP website, which shows the results of 10 different transmembrane domain prediction programs and a consensus created by CCTOP (showed on the first row). Red lines indicate protein regions with a higher probability of residing “inside” the cell (i.e. in the cytosol) and blue lines regions residing “outside”. The grey line at the beginning of PmoB indicates the prediction of a signal peptide. All predictions were scored by the software with high reliability (PmoC: 93.4 %, PmoA: 90.4 % and PmoB: 98.9 %).


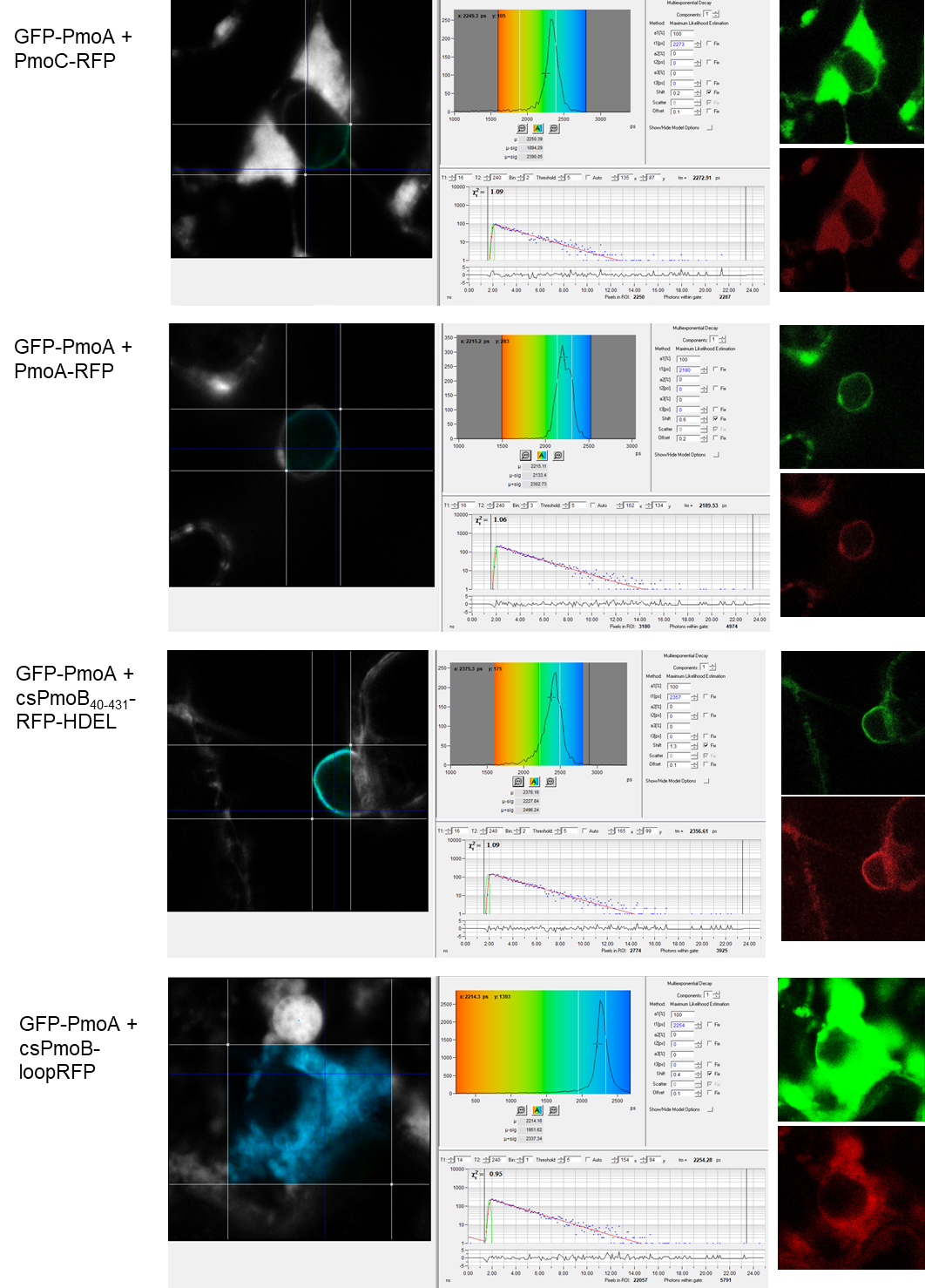


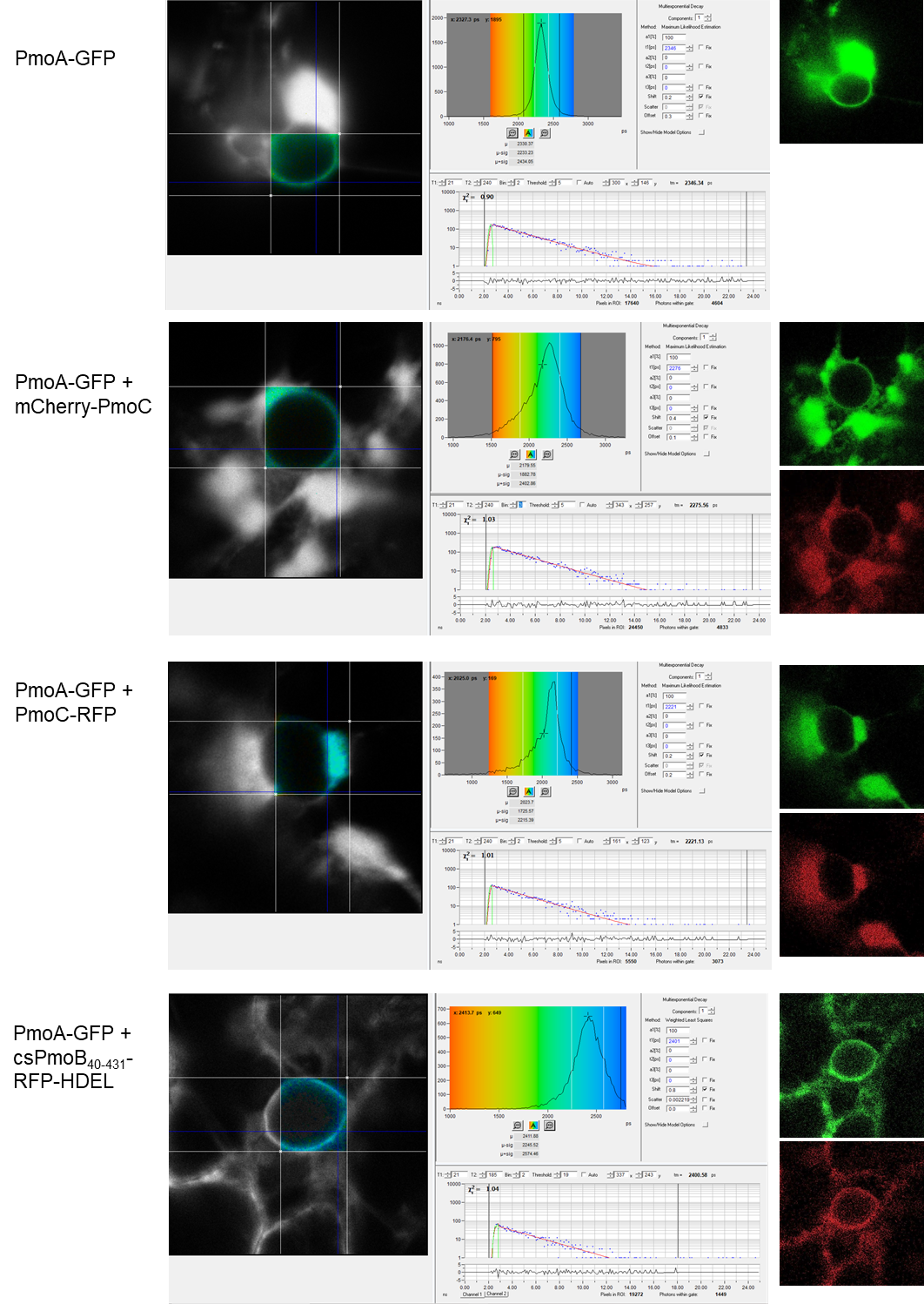


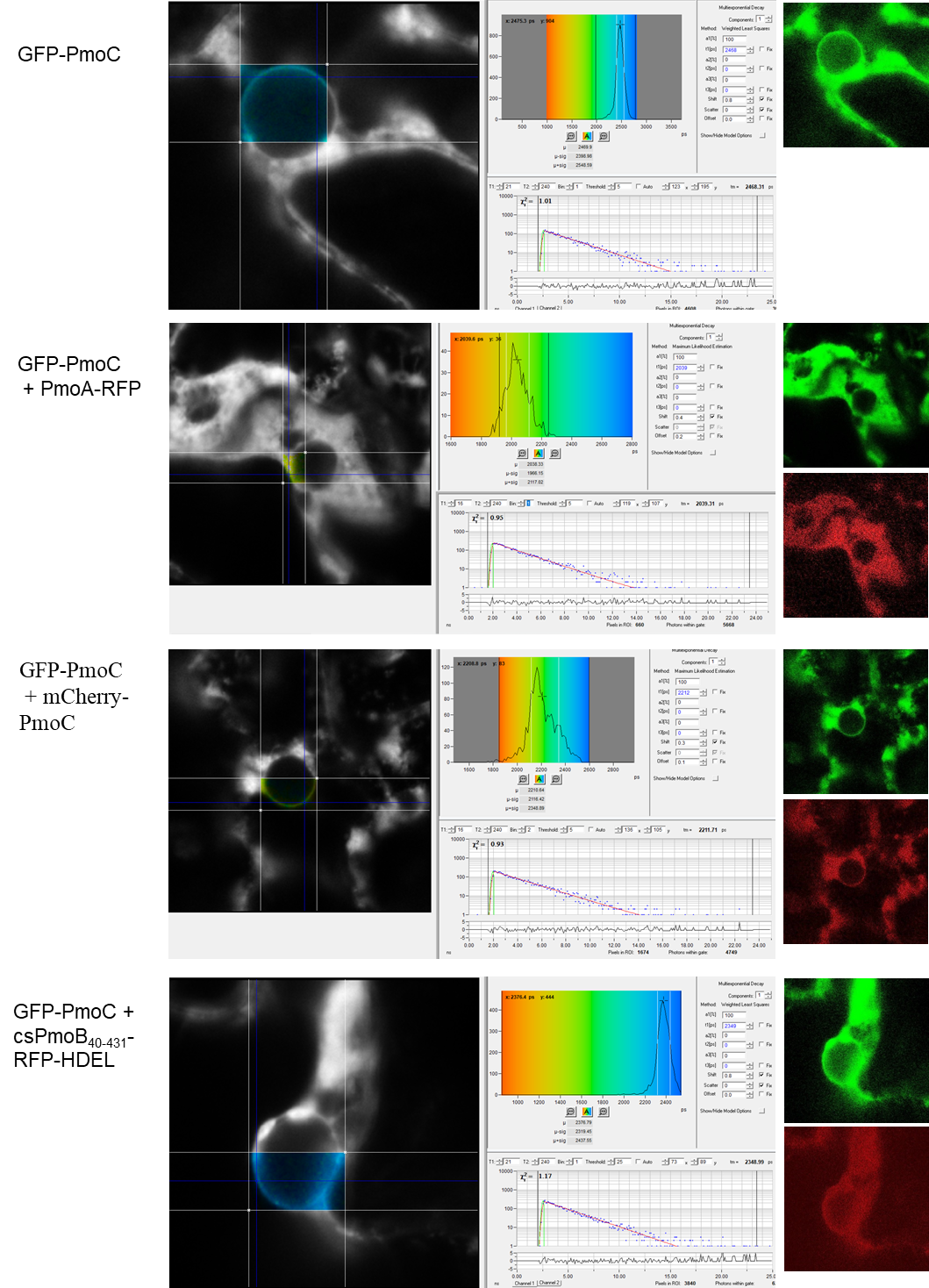


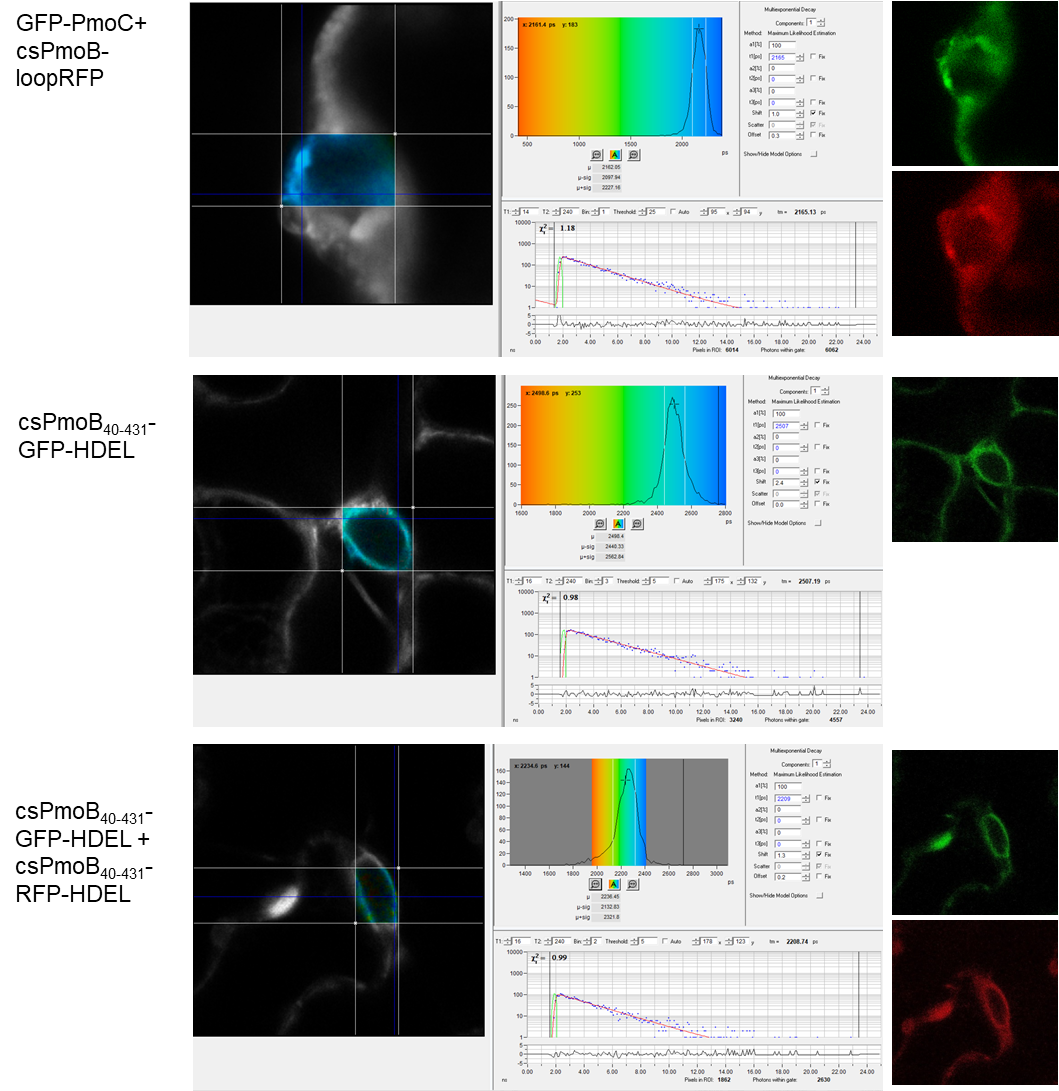


**Supplementary Figure S5.** **Pairwise** **FRET-FLIM analysis of different pMMO subunit interactions.**

Example images of each combination used for FRET-FLIM, showing the selected region of the nuclear envelope for which the chi-squared showed a good fit, the color-coded distribution of the GFP lifetime, the GFP lifetime decay curve for a single pixel within the area and its fit to an exponential curve, and raw images on the right. Images are shown for the donors alone (controls), and in the presence of RFP-tagged acceptors.


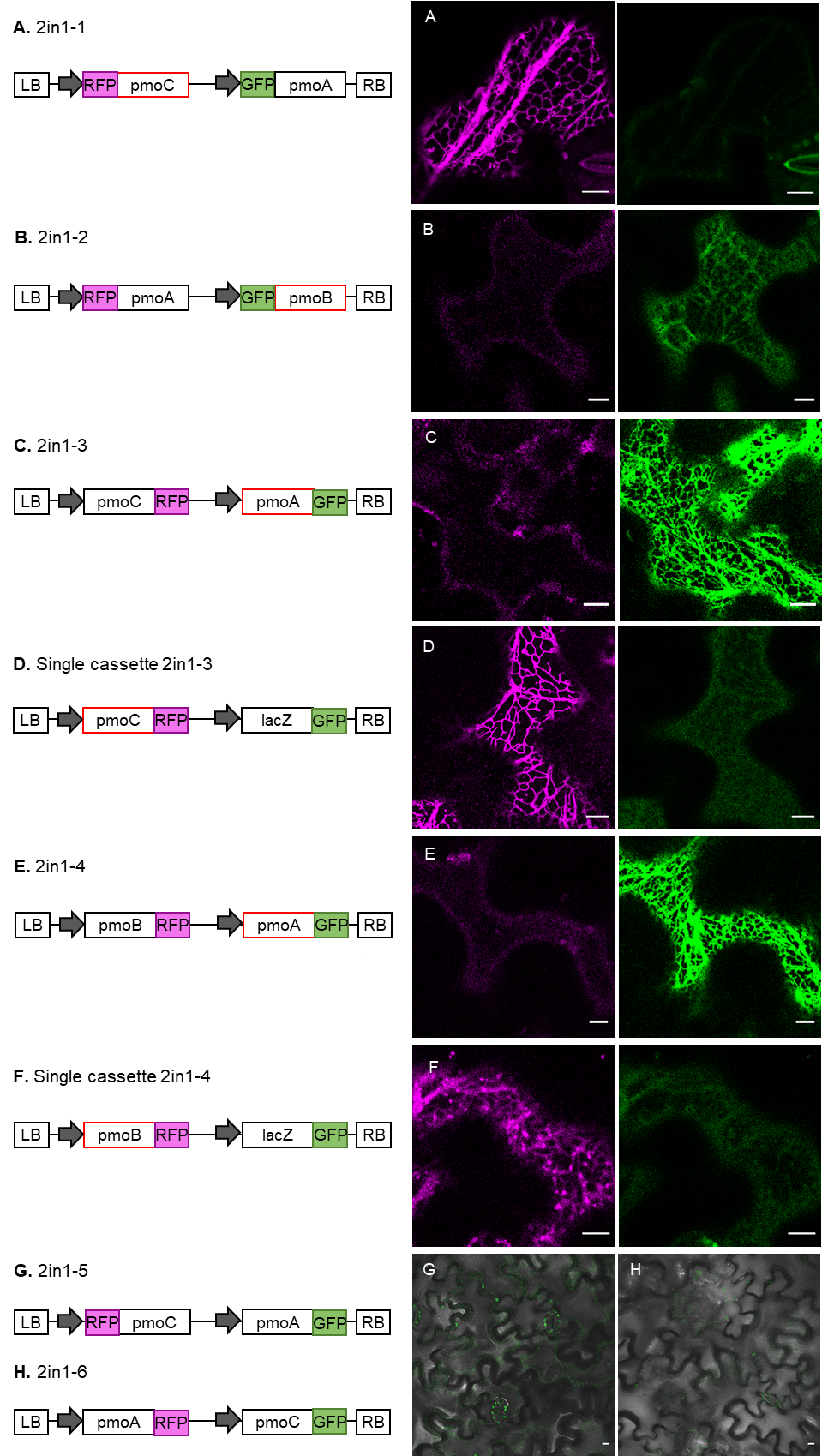


**Supplementary Figure S6. Combinatorial design and expression of 2in1 vectors containing pMMO subunits.**

2in1 vectors contain two cloning cassettes within the same T-DNA flanked by left border (LB) and right border (RB) regions, each cassette driven by a strong constitutive promoter (35S) (grey arrow) and a fluorescent protein in frame to allow the fusion of the gene of interest (Hecker et al., 2015). Transient expression at 2 days in tobacco with cassettes that show expression outlined in red: (A) 2in1-1, (B) 2in1-2, (C) 2in1-3, (D) 2in1-3 with no recombination of the second cassette (“single cassette”), now shows expression of the first cassette, (E) 2in1-4, (F) single cassette 2in1-4 which now expresses the first cassette, (G) and (H) 2in1-5 and 2in1-6 without any expression. Scale bar = 10 μm.

**Supplementary Figure S7. Uncropped DNA gel image from Figure 5C**


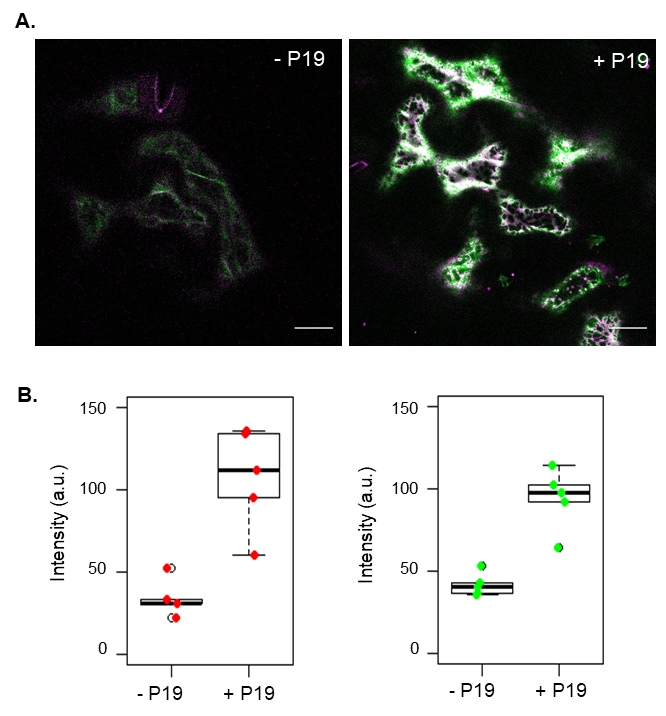


**Supplementary Figure S8. Co-expression of RFP-CABClover with P19.**

(A) RFP-CABClover was expressed in *Nicotiana benthamiana* at 2 days with and without P19, with an increase in expression being observed in both channels with co-expression with P19. (B) The average fluorescence intensity levels were measured using ImageJ in 20 ROIs with 5 biological replicates. On average the RFP intensity from RFP-CABClover without P19 was 34 ± 5 (mean ± s.e.m, n=5) and with P19 was 107 ± 14 (mean ± s.e.m, n=5) measured on an 8 bit depth (0-255). The GFP intensity, from the terminal clover in the polypeptide, increased from an average of 41 ± 3 (mean ± s.e.m, n=5) without P19 to 94 ± 8 (mean ± s.e.m, n=5) with P19. Scale bar = 100 μm.


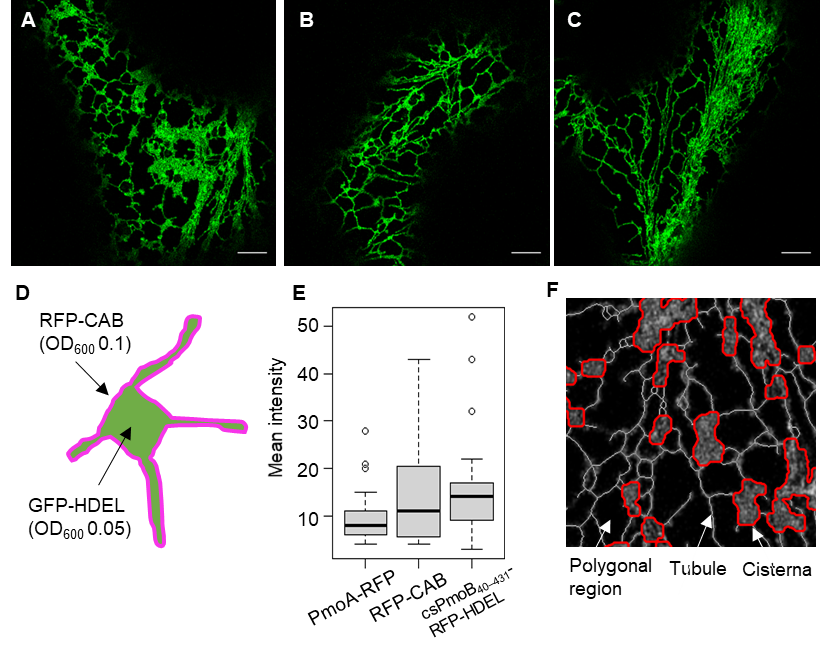


**Supplementary Figure S9. Rationale and design for AnalyzER experiment.**

Airyscan images of tobacco cells expressing GFP-HDEL from the same leaf piece showing different ER morphologies: (A) a high abundance of cisternae, (B) an intermediate proportion of tubules and cisternae, (C) a highly tubular network with a stream of appressed tubules. Scale bar = 5 μm. (D) Schematic diagram showing the co-expression of pMMO proteins on the ER membrane (magenta) with GFP-HDEL in the lumen (green). (E) Quantification using ImageJ of the RFP intensity in the ER of cells used for AnalyzER (n=22 for PmoA-RFP, n=20 for RFP-CAB, and n=27 for csPmoB_40–431_-RFP-HDEL). (F) Example of the skeletonization process using AnalyzER to segment the different ER structures. The AnalyzER software segments the ER network using an intensity-based threshold combined with intensity-independent phase congruency enhancement to include dimmer structures such as thin tubules (Pain *et al.*, 2019). The network is converted into a single pixel-wide “skeleton”, where tubules are represented as lines, and cisternae are defined based on an area cut-off. The software also analyses the morphology of “polygonal regions” which are enclosed between tubules and cisternae.


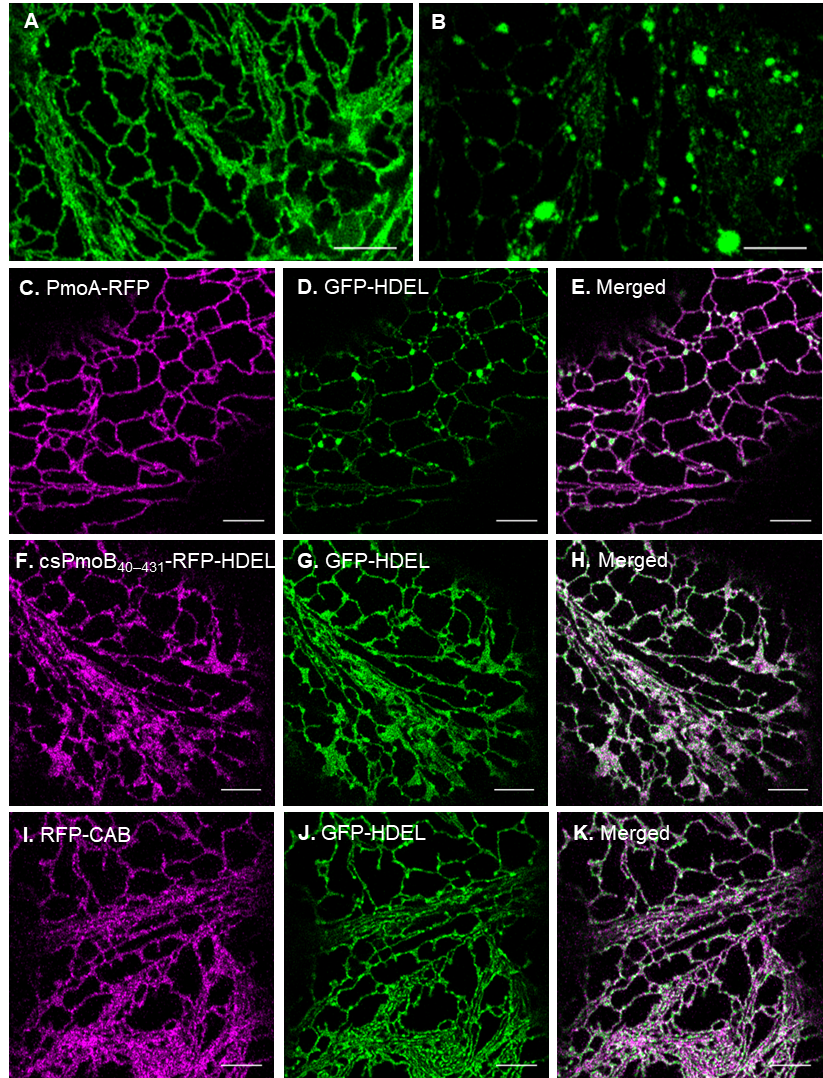


**Supplementary Figure S10. Example images taken for AnalyzER.**

(A) Expression of the GFP-HDEL marker in a control cell, and (B) in a cell co-expressing PmoA-RFP, resembling a constriction phenotype. (C-E) Co-expression of PmoA-RFP with GFP-HDEL, (F-H) co-expression of csPmoB_40–431_-RFP-HDEL with GFP-HDEL, (I-K) co-expression of RFP-CAB with GFP-HDEL. Scale bar = 5 μm.


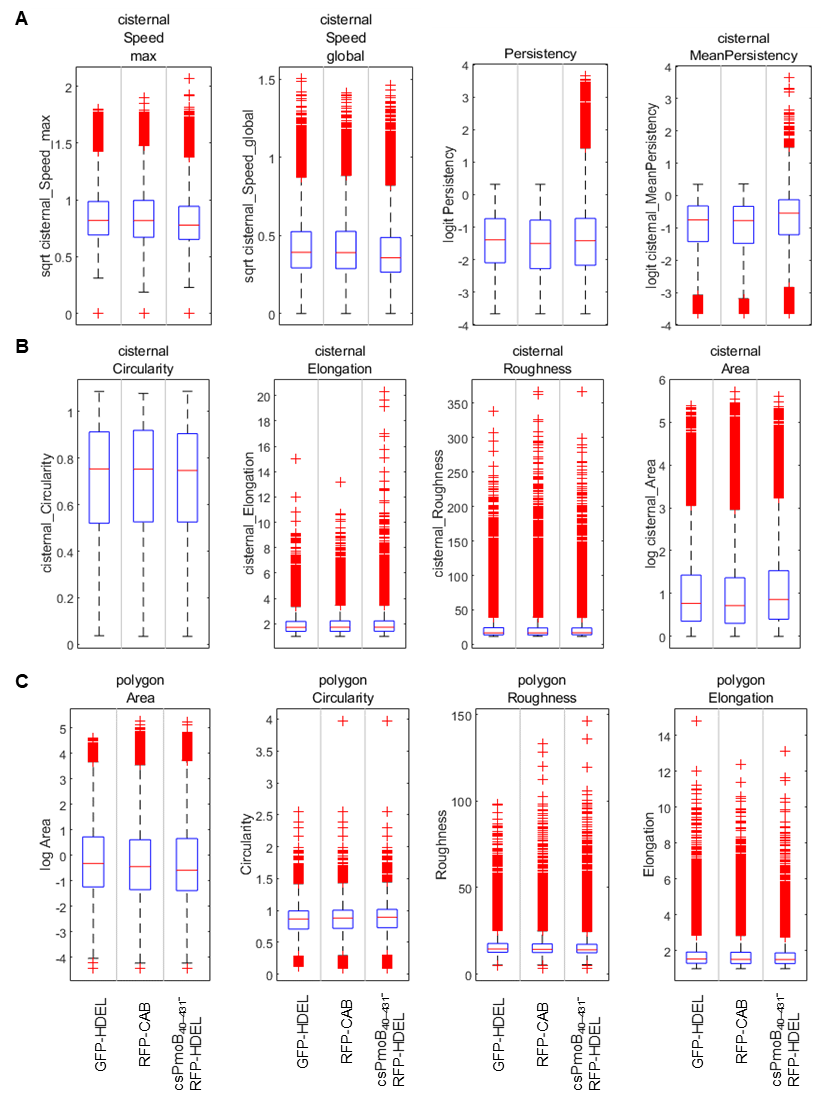


**Supplementary Figure S11. Metrics of ER dynamics and morphology.**

(A) Boxplots of cisternal maximum and average speed, and of tubule mean persistency and cisternal mean persistency. (B) Boxplots of cisternal morphology metrics (circularity, elongation, roughness and area). (C) Boxplots of morphology of polygonal regions (circularity, elongation, roughness and area). RFP-CAB + GFP-HDEL (n = 20), csPmoB_40–431_-RFP-HDEL + GFP-HDEL (n = 27), and GFP-HDEL alone (n = 26). Boxplots show the median (centre line), upper and lower quartiles (box limits), 1.5x interquartile range (whiskers), and outliers (red crosses).


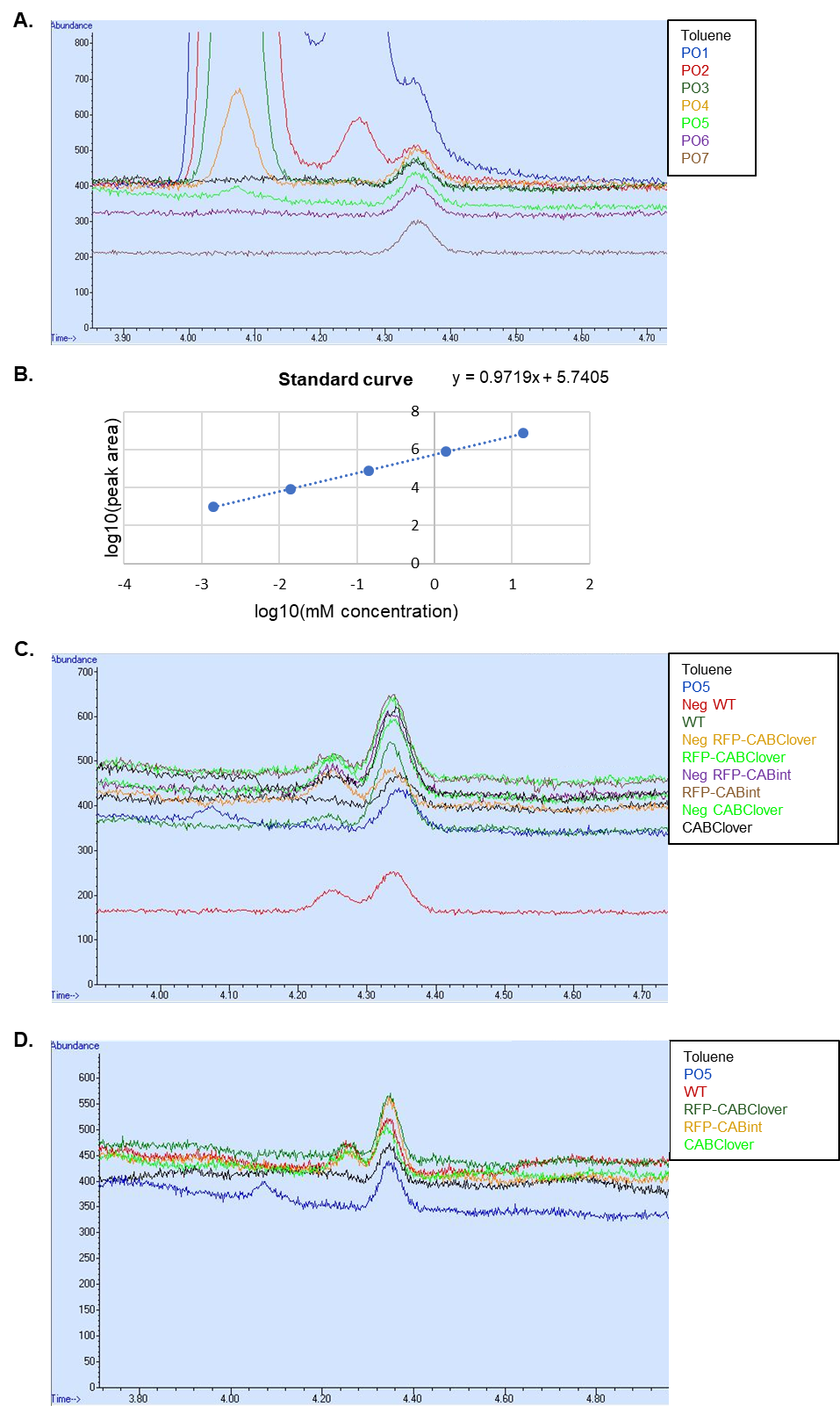


**Supplementary Figure S12. pMMO propylene epoxidation assay.**

(A) Propylene oxide (PO) standards with the solvent (toluene) were prepared as 1:10 serial dilutions (PO1: 14 mM to PO7: 1.4 x 10^-5^ mM). The curves show the PO peak (58 m/z) appearing with a retention time of 4.073 seconds. PO5 (0.0014 mM) is the lowest dilution in which a peak is detected, which equates to a 0.08 μg ml^-1^ detection limit of the system for PO. (B) Standard curve of the log10(peak area) as a function of the log10(concentration) for the 5 dilutions in which PO can be detected. (C) Curves for PO for the blank (toluene – black), PO5 (blue), and plant lysates incubated for 10 minutes at 30 °C. Negatives are samples incubated without propene. (D) Curves for PO for toluene, PO5 and plant lysates incubated for 30 minutes at 30°C.

**Supplementary Table 1. Fluorescence lifetimes in FRET-FLIM analysis.**

Interactions between the pMMO subunits were analysed. Donor and acceptor protein constructs are listed together with the average fluorescence lifetime (in ns) for the donor fluorophore and the SD for each combination. The difference between control and test samples was calculated (Δ). It was previously reported that a reduction in excited-state lifetime of 0.2 ns is indicative of energy transfer (Stubbs et al., 2005). For each combination, at least three biological samples with a minimum of six cells were used for the statistical analysis.

| **Donor (GFP)** | **Acceptor (RFP)** | **Average [ns] ± SE** | **Δ [ns]** |
| --- | --- | --- | --- |
| GFP-PmoA | - | 2.38 ± 0.02 |  |
| GFP-PmoA | csPmoB_40–431_-RFP-HDEL | 2.39 ± 0.01 | 0 |
| GFP-PmoA | mCherry-PmoC | 2.22 ± 0.01 | 0.16 |
| GFP-PmoA | PmoC-RFP | 2.25 ± 0.02 | 0.13 |
| GFP-PmoA | PmoA-RFP | 2.22 ± 0.01 | 0.17 |
| GFP-PmoA | csPmoB-loopRFP | 2.25 ± 0.04 | 0.13 |
|  |  |  |  |
| PmoA-GFP | - | 2.34 ± 0.01 |  |
| PmoA-GFP | csPmoB_40–431_-RFP-HDEL | 2.40 ± 0.01 | 0.06 |
| PmoA-GFP | mCherry-PmoC | 2.20 ± 0.01 | 0.14 |
| PmoA-GFP | PmoC-RFP | 2.20 ± 0.03 | 0.14 |
|  |  |  |  |
| GFP-PmoC | - | 2.42 ± 0.01 |  |
| GFP-PmoC | csPmoB_40–431_-RFP-HDEL | 2.37 ± 0.01 | 0.05 |
| GFP-PmoC | PmoA-RFP | 2.16 ± 0.03 | 0.26 |
| GFP-PmoC | mCherry-PmoC | 2.19 ± 0.02 | 0.23 |
| GFP-PmoC | csPmoB-loopRFP | 2.19 ± 0.05 | 0.23 |
|  |  |  |  |
| csPmoB_40–431_-GFP-HDEL | - | 2.45± 0.01 |  |
| csPmoB_40–431_-GFP-HDEL | csPmoB_40–431_-RFP-HDEL | 2.31± 0.02 | 0.15 |

**Supplementary Table 2. ANOVA and post-hoc comparisons.**

ANOVA carried out with the same dataset used for MANOVA and boxplots with the associated F-statistic and p-value. Post-hoc Tukey HSD comparisons are shown with significant differences marked as: *p < 0.05, **p < 0.01, and ***p < 0.001.

| Variable | F | p-value | RFP-CAB/  GFP-HDEL | csPmoB_40–431_-RFP-HDEL/  GFP-HDEL | RFP-CAB/  csPmoB_40–431_-RFP-HDEL |
| --- | --- | --- | --- | --- | --- |
| Tubule length | 0.4124 | 0.664 |  |  |  |
| Tubule speed max | 1.998 | 0.143 |  |  |  |
| Tubule speed global | 1.37 | 0.261 |  |  |  |
| Tubule persistency | 1.72 | 0.187 |  |  |  |
| Cisternal contrast | 0.1929 | 0.825 |  |  |  |
| Cisternal correlation | **9.767** | **0.000182** | *** | ** |  |
| Cisternal energy | 0.3012 | 0.741 |  |  |  |
| Cisternal homogeneity | 0.05686 | 0.945 |  |  |  |
| Cisternal speed max | **3.769** | **0.0279** |  | * |  |
| Cisternal speed global | **3.309** | **0.0423** |  |  |  |
| Cisternal persistency | **7.455** | **0.00116** |  | * | ** |
| Cisternal circularity | 0.1594 | 0.853 |  |  |  |
| Cisternal elongation | 0.4431 | 0.644 |  |  |  |
| Cisternal Roughness | 0.6224 | 0.54 |  |  |  |
| Cisternal area | 1.828 | 0.168 |  |  |  |
| Polygon area | 0.1901 | 0.827 |  |  |  |
| Polygon circularity | 2.67 | 0.0763 |  |  |  |
| Polygon Roughness | 1.668 | 0.196 |  |  |  |
| Polygon elongation | 2.663 | 0.0768 |  |  |  |
